# New insights into structural features and optimal detection of circulating tumor DNA determined by single-strand DNA analysis

**DOI:** 10.1038/s41525-018-0069-0

**Published:** 2018-11-23

**Authors:** Cynthia Sanchez, Matthew W. Snyder, Rita Tanos, Jay Shendure, Alain R. Thierry

**Affiliations:** 1 IRCM – Institut de Recherche en Cancérologie de Montpellier, Montpellier, 34298 France; 2 INSERM, U1194, Montpellier, 34298 France; 3 Université de Montpellier, Montpellier, 34090 France; 4 Institut régional du Cancer de Montpellier, Montpellier, 34298 France; 5 Department of Genome Sciences, University of Washington, 3720 15th Avenue NE, Seattle, WA USA

## Abstract

Circulating cell-free DNA (cfDNA) has received increasing interest as an apparent breakthrough approach in diagnostics, personalized medicine, and tumor biology. However, the structural features of cfDNA are poorly characterized. Specifically, the literature has discrepancies with regards to cfDNA size profile. We performed a blinded study of the distribution of cfDNA fragment sizes in cancer patient plasma (*n* = 11), by various ultra-deep-sequencing approaches and quantitative PCR (Q-PCR). Whole-genome sequencing of single-stranded DNA library preparation (SSP-S) revealed that nearly half of the total cfDNA fragment number are below 120 nucleotides, which are not readily detectable by standard double-stranded DNA library preparation (DSP) protocols. Fractional size distribution of cancer patient circulating DNA was very similar using both SSP-S-based or Q-PCR-based methods also revealing that high molecular weight (over 350 bp) cfDNA is a minor component (~2%). These extra small detected cfDNA fragments may mostly result from nicks occurring in blood circulation in one or both DNA strands, which are subsequently revealed through the denaturation step of the SSP and Q-PCR procedures. Detailed analysis of the data suggested that most of the detectable cfDNA in blood has a nucleosome footprint (∼10-bp periodicity repeats). The nucleosome is thus the most stabilizing structure of DNA in the circulation. cfDNA molecules, which are initially packed in chromatin, are released from cells and are then dynamically degraded in blood both within and between nucleosomes or transcription factor-associated subcomplexes. While this study provides new insights into cfDNA size profiles harmonizing sequencing and Q-PCR findings, our data validate the use of a specific Q-PCR method and SSP-S for obtaining an optimal qualitative and quantitative analytical signal.

## Cancer: Short tumor DNA abundant, yet missed by blood tests

Around half of all tumor-derived DNA strands found in the bloodstream of cancer patients are too short for detection by the most commonly used diagnostic sequencing methods. Alain R. Thierry from the Montpellier Cancer Research Institute, France, and colleagues quantified the size distribution of circulating cell-free DNA (cfDNA) using two genomic protocols that analyze single-stranded DNA—and can thus pick up small DNA fragments that are missed by conventional double-stranded approaches. Looking in the blood of patients with tumors of the colon, lung, breast and liver, the researchers showed that nearly half of all stretches of cfDNA are shorter than 120 nucleotides long. These sequences likely result from tightly packed DNA that’s dynamically degraded into smaller and smaller fragments. The findings highlight the need for whole-genome sequencing or other single-stranded methods to get an accurate read on cfDNA profiles.

## Introduction

The discovery of small amount of DNA circulating freely in human blood^1^ led to growing scrutiny with regards to its potential use in various clinical fields.^1–5^ Circulating cell-free DNA (cfDNA) analysis is currently applied in prenatal diagnosis^6^ and shows potential for clinical use in other fields including organ transplant, autoimmune diseases, trauma, myocardial infarction, and sepsis.^1–3^ CfDNA concentration is significantly increased in cancer subjects^7,8^ and a significant proportion of cfDNA may derive from the tumor, providing diagnostic and prognostic potential information. Therefore, the characterization of genetic and epigenetic alterations of tumor cells may be obtained in a non-invasive way by analyzing cfDNA from the plasma or serum of cancer subjects.^3,8^ Thus, detection of mutations leading to resistance to targeted therapies, personalized therapeutic monitoring, and non-invasive follow-up of the disease may be possible in the course of cancer management care.

While cfDNA analysis appears clinically useful for several pathologies and specific physiological conditions, a precise understanding of its origins and nature, including the size distribution of cfDNA fragments, has not been established.^1^ Nevertheless, the link to histones has been well established by various reports ^9–13^. That said, in the oncology field it is imperative to have detailed information about the size distribution of cfDNA fragments, to examine the maximum cfDNA concentration and to obtain a high level of sensitivity and specificity, especially when detecting rare genetic alterations. Excluding mass spectrometry, all current methods of cfDNA analysis, including sequencing or PCR-based methods, require cfDNA size definition. It has to be noted that for non-invasive prenatal testing several methods have been described for fetal fraction determination that do not need size distribution, for example, SeqFF and Sanefalcon.^14^

CfDNA structure and size depend on the mechanisms of cfDNA release from cells. While we do not know their respective proportions with regards to cfDNA amount shed into the bloodstream, several have been proposed, including necrosis, apoptosis, phagocytosis, active release, and exosome/microparticle release.^1^

In addition to the diversity of cfDNA release mechanisms in the literature, discrepancies in cfDNA fragment size distribution in healthy individuals or cancer patients are apparent. The most reported size distribution is dominated by mononucleosomes and oligonucleosomes,^9^ and has become the basic premise concerning the structure of cfDNA for many years.^1,10^ In particular, conventional next-generation sequencing (NGS) methods, as well as earlier works based on electrophoretic mobility, such as PAGE or the Agilent platform, have revealed clearly a high proportion of cfDNA fragments ranging from 170 to 200 bp in both healthy or cancer individuals.^11,12^ Apoptotic DNA cleavage produces a characteristic pattern ladder of 180–200 bp, or multiples thereof (oligonucleosomes). DNA wrapped around the histone octamer is 147 bp in length and the linker DNA is 20–90 bp (mainly, 20 bp). Association of these fragments with nucleosomes presumably assures structural integrity by protecting DNA from enzymatic degradation in the circulatory system.^13,15,16^ Contrarily, other studies have shown the presence of large-sized fragments of many kilobases (kbp), which may indicate a necrotic release mechanism.^17^ However, these observations should be taken with caution because of the uncertainty of pre-analytical factors, especially with regard to the contamination of DNA derived from blood-cell degradation.

In contrast, we have shown by alternative methods including a Q-PCR-based method and atomic force microscopy (AFM), the majority of cfDNA in cancer patients is <145 bp.^8,18^ By amplifying DNA sequences of increasing size within the same DNA region, Chan et al.^19^ and Diehl et al.^4^ demonstrated the presence of a significant fraction of short cfDNA fragments below 180 bp in healthy individual and cancer patient cfDNA, respectively.^18^ We later showed, for the first time, that the shortest is the amplicon (down to 60 bp), while the highest is the quantification in either healthy or cancer subjects,^8,18,20^ and that mutant cfDNA fragments are shorter than wild-type fragments.^20^ Moreover, this observation suggested that the detection of amplicons <100 bp is more relevant for optimally quantifying cfDNA.^8^ This has been confirmed and now most of the Q-PCR-based methods involve the amplification of DNA sequences <100 bp, while targeting ~150 bp length sequences is known to be optimal for quantifying non-fragmented genomic DNA.^21–23^

CfDNA size distribution obtained by Q-PCR has shown high discordance with other methods, in particular conventional NGS, which precludes drawing any general conclusions. This issue therefore appears critical in respect to diagnostic performance (especially in oncology when testing for genetic alterations, which may be of rare frequencies among cfDNA fragments) as it relies on the number of examined copies. Our two groups pooled our respective expertise in deep-sequencing and Q-PCR methods, which are poorly associated in the literature, to determine in a blinded study the cfDNA size profile in plasma DNA extracts of cancer patients and evaluating performance of various methodologies of both approaches to optimally recover cfDNA copies.

## Results

Double-strand library preparation (DSP) is used typically for cell-free DNA analysis for several reasons: it is an easy protocol to carry out in the laboratory as it takes only a few hours compared to single-strand library preparation protocols, which typically take much longer; it is cheap on a per-sample basis; and a lot of optimization has been done on DSP so that it is more efficient and has less adapter ligation bias.^24^ Single-strand library preparation (SSP) was recently designed to bypass the limits of the conventional DNA library in order to recover damaged and short double-strand DNA fragments, especially in the paleontology field.^25,26^ High-throughput sequencing of cfDNA from SSP (SSP-S) has been used only by Snyder et al.^27^ and Burnham et al.^28^ In this study, we compared the cfDNA size profile obtained, on one hand, by sequencing from DSP and SSP and, on the other hand, in a blinded study by comparing SSP-S with a Q-PCR-based method (SI-1).

Although SSP-S and Q-PCR are based on the detection of double-stranded DNA (dsDNA) as the final analytical signal, cfDNA fragment size using these methods is determined from the size of single-strand DNA (ssDNA) fragments resulting from the denaturation step of both SSP and Q-PCR processes. Consequently, the number of nucleotides (nt) is used as the fragment size distribution length unit when using SSP-S or Q-PCR.

### Size profile obtained from DSP-S of plasma from two cancer patients

**Fig. 1:**
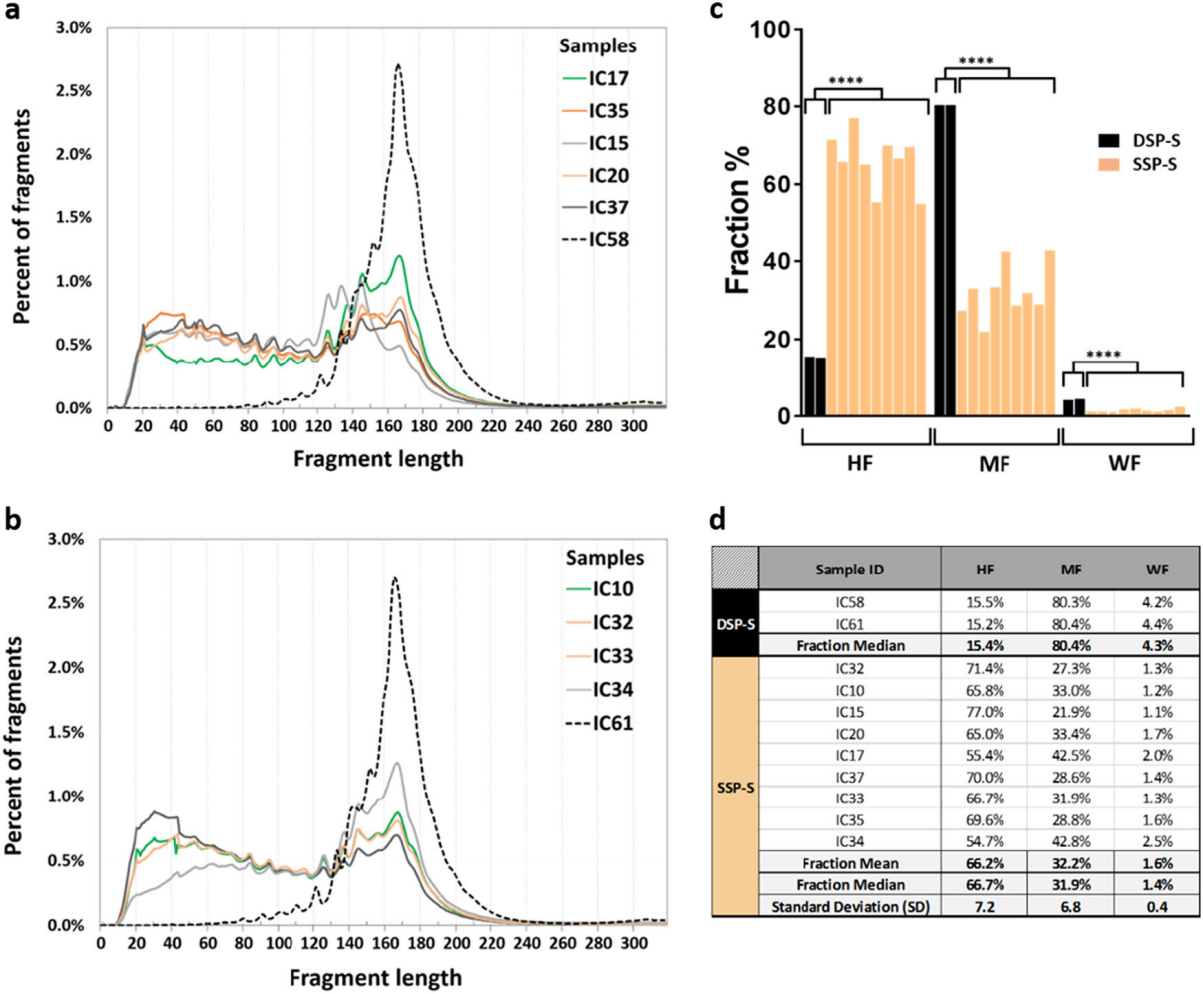
CfDNA fragments shorter than 100 nt are accessible for sequencing following single-strand library preparation (SSP). Comparison of the size profiles of cancer patient cfDNA obtained from DSP-S (dotted lines) as compared to SSP-S (full lines) DSP-S was performed on plasma from lung cancer (IC58 and IC61), while SSP-S was performed on four lung (IC10, IC32, IC15, and IC20), two colorectal (IC33 and IC37), two breast (IC34 and IC35), and one liver (IC17) cancers. In order to differentiate SSP-S derived size profile curves, two sets of data are presented (IC15, IC17, IC20, IC35, IC37, **a**; and IC10, IC32, IC33, IC34, **b**) each containing one of the two cfDNA size profile as determined by DSP-S. The nine cfDNA extracts from cancer patients analyzed by SSP-S were examined in the blinded study comparing SSP-S with Q-PCR size profile analysis. **c** presents the proportion of three size ranges we set based on the previous cfDNA sizing biological paradigm: MF, corresponding to the length of the DNA sequence compacted in a mononucleosome (145–249nt); WF, corresponding to weakly fragmented cfDNA ( 249nt); and HF, corresponding to highly fragmented cfDNA (<145nt). **d** presents the percent value of the three size ranges obtained from each patients either by SSP-S or DSP-S. ****, p < 0.0001

DSP library was sequenced to 96-fold and 105-fold coverage (1.5 and 1.6 billion fragments). DSP-S analysis of two lung cancer patient plasma samples (IC58 and IC61) resulted in very similar size profiles (Fig. 1a, b, respectively). Both showed a Gaussian cfDNA size distribution ranging from 100 to 240 bp, peaking at 166 bp with a half peak width between 150 and 200 bp. Series of smaller peaks at a periodicity of ~10 bp could be observed between 81 and 166 bp in both samples at exactly the same peaking sizes (Fig. 1a, b and Table 1a). The proportion of fragments below 145 bp and over 180 bp are 18.5% and 24%, respectively. DSP-S did not reveal a significant amount of fragments lower than 80 bp.

**Table 1:** Detailed characterization of the ~10 nt periodicity subpeaks observed from size distribution of cfDNA of cancer patients as determined by DSP-S and SSP-S

a												
	Subpeak corresponding size											
	SSP-S (nt)									DSP-S (bp)		Mean SSP-S minus mean DSP-S
Peak	IC17	IC35	IC15	IC10	IC20	IC32	IC33	IC34	IC37	IC 58	IC61	
1	42	43	41	41	42	42	43	43	42	–	–	
2	53	52	52	53	52	52	52	53	52	–	–	
3	62	63	61	62	61	63	60	63	60	–	–	
4	73	72	73	74	73	71	73	73	73	–	–	
5	83	83	83	83	83	83	83	83	83	81	81	2.0
6	94	94	94	94	94	94	94	94	94	92	92	2.0
7	104	104	104	103	104	104	103	103	103	102	102	1.6
8	114	114	114	114	114	114	115	114	114	111	111	3.1
9	125	126	126	125	125	125	125	126	125	122	122	3.3
10	137	136	133	137	136	137	137	137	136	134	134	2.2
11	145	146	144	145	145	145	145	145	145	145	142	1.5
12	156	152	–	156	156	157	156	156	–	152	152	3.6
13	166	165	166	167	167	166	167	166	166	166	166	0.2

b											
Periodicity	Subpeak periodicity										
	SSP-S (nt)									DSP-S (bp)	
	IC17	IC35	IC15	IC10	IC20	IC32	IC33	IC34	IC37	IC 58	IC61
(1–2)	11	9	8	12	7	10	9	10	7	–	–
(2–3)	9	11	9	9	9	11	8	10	8	–	–
(3–4)	11	9	12	12	12	8	13	10	13	–	–
(4–5)	10	11	10	9	10	12	10	10	10	–	–
(5–6)	11	11	11	11	11	11	11	11	11	11	11
(6–7)	10	10	10	9	10	10	9	9	9	10	10
(7–8)	10	10	10	11	10	10	12	11	11	9	9
(8–9)	11	12	12	11	11	11	10	12	11	12	12
(9–10)	12	10	7	12	11	12	12	11	11	12	12
(10–11)	8	10	11	8	9	8	8	8	9	11	8
(11–12)	11	6	–	11	11	12	11	11	21	7	10
(12–13)	10	13	–	11	11	9	11	10		14	14

a, DNA size corresponding to the periodic subpeaks for each patient. Peak numbers are ranked from the smallest to the largest size as observed by SSP-S and DSP-S expressed as nt and bp, respectively. b, Characterization of the fragment periodicity as determined by the lengths between two consecutive subpeaks, which are observed in the size profile of each cfDNA plasma extracts. DNA lengths obtained by SSP-S and DSP-S are expressed as nt and bp, respectively. (–), not detected

### Size profile obtained from SSP-S of plasma from nine cancer patients

SSP library were sequenced to 30-fold coverage (779M fragments). Nine samples showed similar cfDNA size profile using SSP-S. The size distribution was non-Gaussian with two apparent populations: the first ranging between 30 and 120 nt and the other between 120 and 220nt (Fig. 1a, b). The proportion of fragments below 145 bp and over 180 bp are 66.2% and 7.6%, respectively. Whereas size profile by SSP-S peaked at 166–168nt correspondingly to the peak size observed by DSP-S (166 bp), the shorter-length cfDNA fragment population was unique to SSP-S as compared to DSP-S (Fig. 1a, b). Series of smaller peaks at the periodicity of ~10 nt is detected from 41 to 167 nt when using SSP-S, while the same periodicity could be observed only from 81 to 166 bp when using DSP-S (Table 1a, b). The values of this small periodic peak size are strikingly equivalent between the nt and bp number as obtained from SSP-S and DSP-S, respectively (Table 1b). While maximum peak size is identical not only among all tested samples by either SSP-S or DSP-S, the ~10 nt subpeaks revealed by SSP-S corresponded to lengths being 2 to 4 nt shorter than that obtained by DSP-S (Table 1a). Note, size profile pattern among those nine samples as determined by SSP-S showed very high consistency as it exists likewise among the two previously tested samples by DSP-S as well as those previously assessed.^11–13,27^ Note, the fifth and the sixth subpeaks showed exactly the same corresponding size following either SSP-S (83 and 94nt, respectively) or DSP-S (81 and 92 bp), and second, the periodicity between both subpeaks is identical following SSP-S and DSP-S analysis (11nt and 11 bp, respectively). No peak is detectable at lengths superior to 260 nt by SSP-S, while a weak DNA fragment sub-population (<3% of total reads) peaking at 307 and 308 bp (IC58 and IC61, respectively), which may correspond to the size of DNA contained in a dinucleosome, is detectable by DSP-S (SI-2). It should be noted that DSP-S was performed on plasma from lung cancer, while SSP-S was performed on four lung (IC10, IC32, IC15, and IC20), two colorectal (IC33 and IC37), two breast (IC34 and IC35), and one liver (IC17) cancers. Use of SSP generates higher recovery of fragments below 130 bp and the observation of fragments below 80nt as compared to DSP irrespective of the various types of cancer. In addition, CfDNA size profile obtained by SSP-S and DSP-S showed clear discrepancies, especially when comparing fragment size distribution by three size ranges we set based on the previous cfDNA sizing biological paradigm: MF, corresponding to the length of the DNA sequence compacted in a mononucleosome (145–249nt); WF, corresponding to weakly fragmented cfDNA (>249nt); and HF, corresponding to highly fragmented cfDNA (<145nt) (Fig. 1c, d). SSP-S showed, as compared to DSP-S, much higher HF fraction proportion (66.7% median±7.2SD and 15.4% median, respectively; *p* = 5,41.10-6), and lower MF (31.9% median ± 6.8 SD and 80.4% median, respectively; *p* = 5,33.10-6) and WF (1.4% median ± 0.4 SD and 4.3% median, respectively; *p* = 1,74.10-5) proportion. Note, HF, MF, and WF proportion are rather homogeneous for either SSP-S (0.9095; 0.9098; 0.883 *R*^2^, respectively; Fig. 1c, d) or DSP-S (Fig. 1c, d).

### Blinded study comparing SSP-based sequencing and Q-PCR

**Fig. 2:**
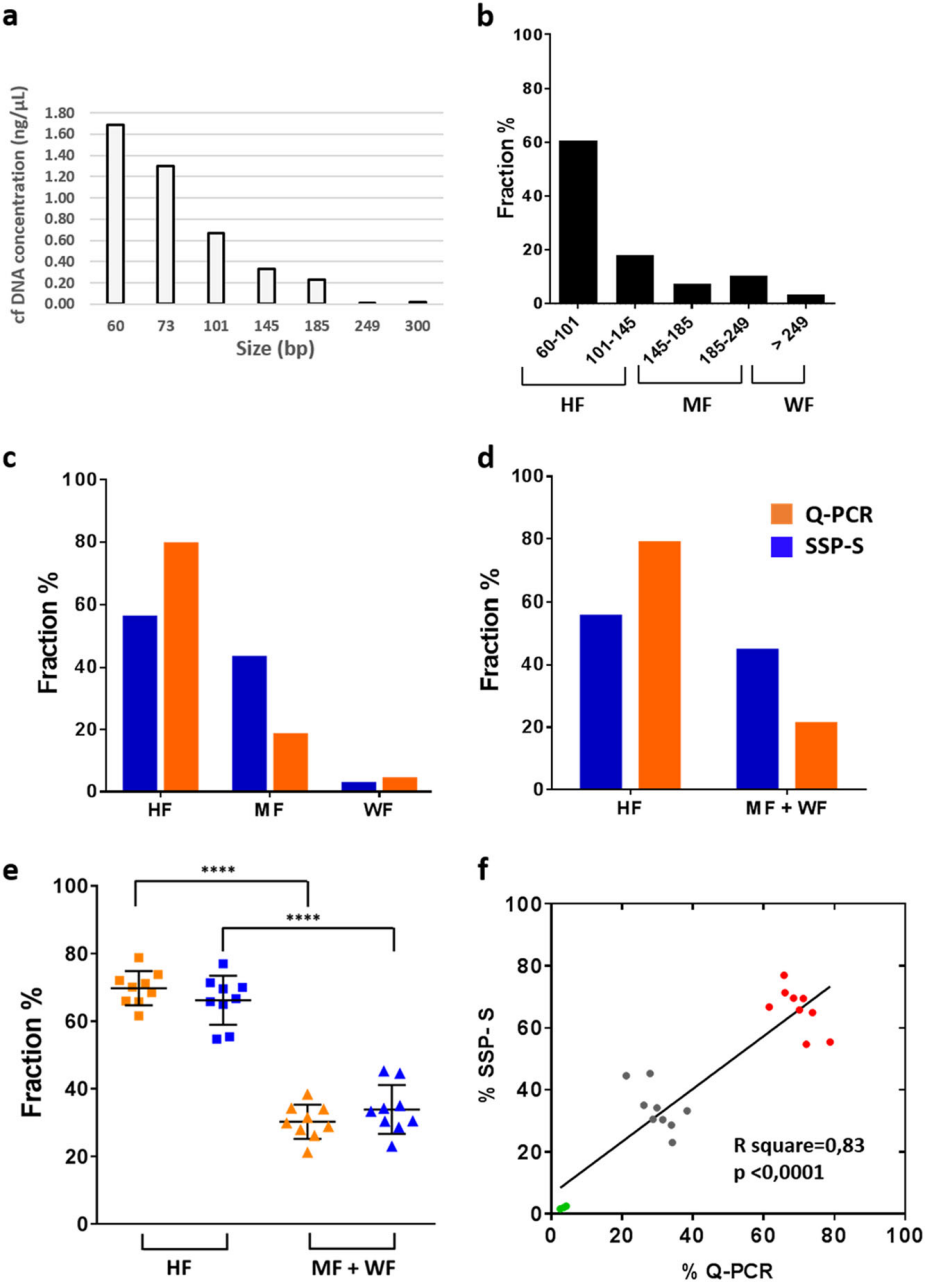
Determination of cfDNA fragment size distribution by Q-PCR from plasma of a liver cancer patient (IC17). **a** Fragment length distribution is obtained by detecting variable amplicon lengths (bp) within the same DNA region. As cfDNA is randomly fragmented in plasma, the fragment amount, as measured by PCR, decreases monotonically with amplicon length, with a gradient that is a function of the underlying fragment length distribution. **b** Fractional size distribution, as determined under Methods Online, corresponds to the proportion of cfDNA per size range as a percentage of the total observed cfDNA by Q-PCR. **c**, **d**, blinded comparison of the fractional fragment size profile obtained by SSP-S (blue) and Q-PCR (orange). HF, highly fragmented DNA (<145nt); MF, mononucleosome fragmented DNA (145–249nt); WF, weakly fragmented DNA (>249nt); MF + WF (>145nt). **e** Combined data from the nine cancer patient plasma examined in the Q-PCR vs. SSP-S comparative study. cfDNA fractional fragment size distribution from cancer patients (*n* = 9) by Q-PCR and SSP-S methods is displayed upon two fractions: HF, highly fragmented DNA (<145nt) and MF + WF (>145 nt). For both analytical approaches, HF fraction is statistically different to the MF + WF fraction when using either Q-PCR or SSP-S (****p = 1.65 × 10^−11^ and, ****p = 5.81 × 10^−8^, respectively). **f** Correlation of the cfDNA fractional fragment size distribution determined by SSP analysis with that obtained from Q-PCR from the same samples of the blinded study (*n* = 9). Green, gray, and red dots correspond to the WF, MF + WF, and HF size fraction, respectively. Due to the low DNA concentrations in some patient plasma samples, cfDNA fractional distribution was not analyzed by all target sequence sizes among the examined samples. Data are expressed as a percentage of the cfDNA fractional fragment size

To shed light on the seemingly divergent findings in the literature, SSP-S and Q-PCR analysis of the same cfDNA extract were compared in a set of nine cancer patient samples (IC10, 15, 17, 20, 32, 33, 34, 35, and 37). The determination of the fractional size distribution using the Q-PCR method is illustrated in Fig. 2 for the IC17 patient as indicated in SI-1 (Fig. 2a–d). The fractional size distribution of all cancer patients is represented in SI-3 and Table 2a. HF, MF, and WF size range proportion values were determined either by SSP-S (SI-4a, SI-5, Fig. 1d) or Q-PCR analysis as illustrated in Fig. 2b–d in case of the IC17 patient. Data obtained from the IC17, IC34, and IC35 patients showed that HF was the main fraction ranging from 55.4% to 69.6% and from 68.5% to 78.8% determined by SSP-S and Q-PCR, respectively; MF was the second main fraction ranging from 28.8% to 42.8% and from 17.7% to 28.9% determined by SSP-S and Q-PCR, respectively; WF was by far the lowest fraction ranging from 1.6% to 2.5% and from 2.6% to 4.3% determined by SSP-S and Q-PCR, respectively (SI-6a, SI-7a). Consistent with the previous observation for IC17, IC34, and IC35, HF and MF + WF values determined by Q-PCR methods in all nine patient plasma tested in blind (IC10, 15, 17, 20, 32, 33, 34, 35, and 37) were similar (HF: 69.8 ± 5.1%) (SI-6b, SI-7b). Fractional size distribution according to HF, MF + WF, or WF determined by SSP-S and Q-PCR correlated significantly (*R*^2^ = 0.83; *p*< 0.0001) (Fig. 2e, f), SI-6, Table 2 and SI-8). The percentage decrease from HF to MF and to WF was similar for both SSP-S and Q-PCR. The fraction decreased by four-fold to two-fold from HF to MF, and by 15-fold to 80-fold from HF to WF. Because of the paucity of the extracted DNA for carrying out Q-PCR analysis, only the HF and MF + WF fractions could be compared for some samples. Fractional size distribution by SSP-S was also determined according to the size of the amplicon length detected by Q-PCR (Table 2b).The cfDNA proportion shorter than 145 nt made up a substantial proportion of cfDNA determined by SSP-S and Q-PCR (66.2 ± 7.2 % and 69.8 ± 5.1%, respectively) (SI-7b). Altogether, the data showed the higher proportion of the fraction below 145 nt (Fig. 2e) and the statistical correlation between value obtained by both methods (Fig. 2f).

**Table 2:** Fractional fragment size distribution of cfDNA of cancer patients by Q-PCR analysis (a) and SSP sequencing (b)

a											
Sample ID	Patient gender	Cancer type	Stage	cfDNA yield (ng/ml)	Fraction						
					60–73	73–101	101–145	145–185	185–249	249–300	>300
IC17	M	Liver	IV	39	23.0%	37.6%	18.1%	7.5%	10.3%	2.7%	0.8%

Sample ID	Patient gender	Cancer type	Stage	cfDNA yield (ng/ml)	Fraction				
					60–101	101–145	145–185	185–249	>249
IC35	F	Breast	IV	16.2	50.1%	18.4%	8.2%	20.7%	2.6%
IC34	F			33.6	66.3%	5.8%	8.2%	15.5%	4.3%

Sample ID	Patient gender	Cancer type	Stage	cfDNA yield (ng/ml)	Fraction			
					60–101	101–145	145–185	>185
IC15	N	Lung	IV	22.5	51.2%	14.6%	15.6%	18.6%
IC37	F	Colorectal		15.9	61.4%	9.8%	4.9%	23.9%

Sample ID	Patient gender	Cancer type	Stage	cfDNA yield (ng/ml)	Fraction		
					60–101	101–145	>145
IC20	M	Lung	IV	21.9	54.7%	19.2%	26.2%
IC32	F			9.6	50.4%	15.7%	34.0%
IC33	M	Colorectal		13.8	61.4%	0.2%	38.4%

Sample ID	Patient gender	Cancer type	Stage	cfDNA yield (ng/ml)	Fraction	
					<145	>145
IC10	F	Lung	IV	11.4	70.1%	29.9%

b											
Origin	Sample ID	Patient gender	Cancer type	Stage	cfDNA yield (ng/ml)	Fraction					
						SSP sequencing					
						30–59	60–100	101–145	146–180	181–249	250–1000
Blind study	IC32	F	Lung	IV	9.6	25.5%	24.0%	21.9%	22.0%	5.3%	1.3%
	IC10	F			11.4	20.5%	22.6%	22.7%	26.5%	6.4%	1.2%
	IC15	M			22.5	19.2%	23.2%	34.6%	18.6%	3.3%	1.1%
	IC20	M			21.9	18.9%	21.4%	24.7%	26.8%	6.5%	1.7%
	IC17	M	Liver		39	12.4%	16.4%	26.7%	35.3%	7.3%	2.0%
	IC37	F	Colorectal		15.9	21.2%	24.4%	24.4%	23.5%	5.1%	1.4%
	IC33	M			13.8	20.3%	23.0%	23.4%	25.2%	6.7%	1.3%
	IC35	F	Breast		16.2	22.7%	22.9%	24.0%	23.7%	5.1%	1.6%
	IC34	F			33.6	12%	18.9%	23.8%	34.5%	8.2%	2.5%
Fraction mean						19.2%	21.9%	25.1%	26.3%	6,00%	1.6%
Standard deviation (SD)						4.4	2.6	3.7	5.5	1.4	0.4

The same DNA extract was analyzed by both methodological approaches under blinded conditions. Data are expressed as percentage of the cfDNA size fraction

### Post hoc cfDNA size profile examination of cancer patient plasma by Q-PCR analysis

In order to consolidate our observations on cfDNA size distribution by Q-PCR, we enlarged the number of cancer patients by including an additional seven plasma samples (SI-9 and SI-10). All seven plasma DNA exhibited similar size profiles, which were equivalent to the nine samples tested in the blinded study (SI-4b). A higher proportion of cfDNA lower than 145 nt was observed in these additional plasma samples. The 16 samples of the blinded study (*n* = 9) and the post hoc study (*n* = 7) showed size profile homogeneity with HF and MF + WF size fractions of 71.6 ± 5% and 28.4 ± 5%, respectively (SI-4b and SI-8a).

### Estimation of average DNA molecule length

Estimation of average DNA molecule length was analyzed by the method of Deagle et al.^29^ The mean fragment size for IC17, IC35, IC34, IC15, IC37, IC20, IC32, IC33, IC10, and IC104 was found as 63, 83, 83, 77, 91, 63, 77, 91, 71, and 62 nt, respectively (SI-11a). A correlation of the average fragment size determined by the Deagle et al.^29^ method and the proportion of cfDNA MF + WF fraction was found (SI-11b).

### Study of the efficacy of targeting short sequences with respect to fragment size

By analyzing PCR with agarose gel electrophoresis, we showed that targeting a DNA sequence of the same size or longer than the input DNA fragment produced a similar PCR yield (SI-12). This demonstrates that efficacy of PCR targeting short DNA sequence is not restricted when the starting material contains short fragments equal or close to the amplicon size, and that does not lead to bias by favoring the amplification of longer fragments.

## Discussion

When directly comparing the cfDNA size profiles of cancer patient cfDNA extract obtained by SSP-S and DSP-S, a high discrepancy was found. The population corresponding to the DNA size wrapped around a mononucleosome (120–220-bp range, peaking up to 167 bp) was observed using both methods. Such a profile was analogous to that of several reports analyzing cfDNA^11,12,27^ and the plasma of pregnant women or organ transplant recipients,^6^ suggesting DNA fragmentation during apoptosis.^9,17^ However, SSP-S revealed a substantial cfDNA fragment population ranging from 30 to 130 nt, especially from 30 to 80 nt, which was not detectable using the DSP library. CfDNA appeared more accessible for sequencing following SSP-S, as previously reported by our group^27^ and then by Burnham et al.^28^ It should be noted that the degree of depletion of short DNA molecules using double-stranded library preparation can be different across different studies, which can be due to adaptors clean-up steps. Fragments shorter than 100 nt are in abundance in cancer patient-derived plasma, but conventional DSP-S methods appeared insensitive to ultra-short cfDNA, emphasizing the need to use SSP-S for optimally examining cfDNA profiles. The SSP library has been recently described and used to generate high-resolution genomes when examining paleontological ancient DNA.^25,26^ This method uses a single-strand DNA ligase and a 5′-phosphorylated and biotinylated adapter oligonucleotide to capture and bind single-strand DNA molecules to beads without prior end repair.^25^ dsDNA is generated through use of primers from this ligation, and subsequently receives a second adaptor via blunt-end ligation. Completion of the adaptor sequence through an amplification reaction is then carried out from finished single strands obtained by heating the previously obtained molecules.

**Fig. 3:**
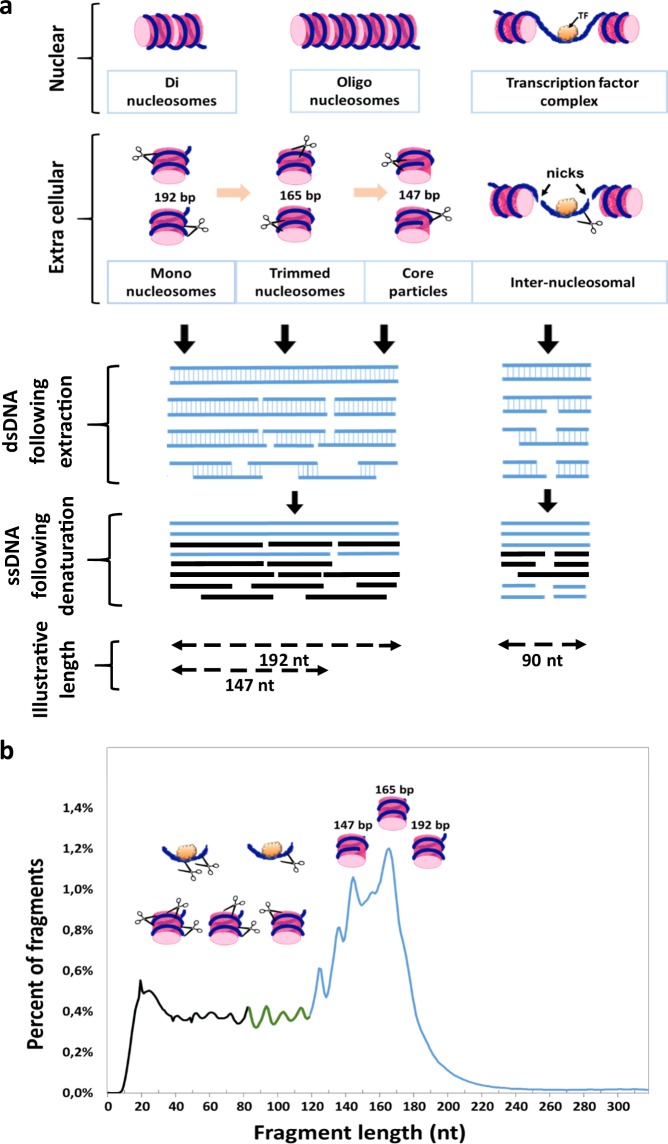
Schematic diagrams of circulating DNA fragmentation from nucleosomes. **a** Two hypotheses are presented: DNA wrapping around a histone octamer or bound to transcription factor (TF). Different types of dsDNA fragments are schematically represented and may exhibit nicks. DNA double-strand or single-strand breaks may occur inside or outside both types of DNA/protein complexes, inside or outside cells. Following DNA denaturation (such as under PCR or SSP preparation), the resulting single strands may be of varying size from several oligonucleotides to few hundreds of nucleotides. The lengths given are indicative. They are based on nucleosome consisting of an octamer of core histone proteins wrapped ~1.65 times by 147 bp of DNA and on the presence of linker DNA describing the non-nucleosomal DNA connecting two or more nucleosomes in an array with length ranging between 20 and 90 bp and varying among different species, or tissues. SsDNA fragments produced by SSP or Q-PCR are subsequently replicated, and sequenced or quantified by SSP-S or Q-PCR, respectively. **b** cfDNA structures and fragmentation with regards to size profile as determined by SSP-S of cfDNA extracted as illustrated from the IC17 patient. Three fractions of the size profile could approximately be distinguished in light of our observations and other works:^12,13,16,27^ Blue curve, DNA fragments originating from cfDNA packed within mononucleosome without any intranucleosomal nicks revealed by both SSP-S and DSP-S; green curve, DNA fragments originating from cfDNA packed within mononucleosome exhibiting nuclease nicks, or within TFs without any nicks, observed by both SSP-S and DSP-S; and black curve, DNA fragments originating from cfDNA packed within mononucleosome with more nicks or within TFs with nicks, which are only observed by SSP-S

SSP-S offers various advantages over DSP-S with regard to the detection of cfDNA. The two most important reasons seem to be the different ligases used by each protocol and the denaturing step in the single-strand preparation. First, circligase, used in the SSP, is more efficient on small fragments than on longer fragments, and is almost certainly more efficient than T4 DNA ligase for short cfDNA fragments. Second, fragments that are damaged, for example, with nicks or abasic sites, are likely to be lost during DSP, but are retained during SSP, so if a sample has DNA damage (e.g., cfDNA), SSP is likely to capture the damaged molecules. DNA molecules with single-strand breaks on one or both strands may be present in cfDNA (Fig. 3). Whereas such molecules are completely lost under DSP procedure, SSP results to DNA break down into several fragments during heat denaturation and each fragment has an independent chance of being recovered in the library.^26^ Third, through the initial biotinylation of cfDNA, all SSP reaction steps are performed while the DNA is tightly bound to the streptavidin-coated beads.^26^ Loss of molecules in the DNA purification steps using silica spin columns or carboxylated beads, which are integral parts of DSP methods, are avoided. Fourth, DSP requires multiple bead-based, size-selective steps eliminating unwanted adapter-dimer products, whereas SSP does not require size-selective steps that eliminate shorter fragments.^28^ Consequently, SSP libraries may contain a larger fraction of shorter molecules than those produced by the double-strand method as demonstrated by Bennett et al.^25^ They observed that SSP improved the recovery of a higher proportion of mapped reads at almost every bin size, which could decrease for increasing fragment lengths although this is still controversial in the literature.^25^ Hence, we cannot totally rule out that SSP-S enriched short over longer fragments and might generate a bias in the representation of the natural distribution.

Our blinded study using a Q-PCR method^18,20^ on the same DNA extracts used for SSP-S showed strikingly similar fractional size distribution as those obtained using SSP-S. We previously showed using the Q-PCR method^30^ that size distribution of cfDNA fragments is significantly lower than the conventional paradigm wherein the lowest size is that of the DNA sequence wrapped around a single histone octamer (147–200 bp, 180 bp mean).^18,20^ Here, direct comparison of SSP-S and Q-PCR analysis showed a higher distribution of cfDNA fragments lower than 145 nt (145 bp corresponds to DNA wrapped around a nucleosomal core unit (167 bp) minus a linker fragment DNA of ~20 bp). Q-PCR is a robust and validated technique, but a few reports have scrutinized its efficiency and variation in quantifying short DNA fragments^25,31^ (SI-12). We demonstrated in this study that our fractional size profile determination by Q-PCR assay does not show any bias in artificially enriching short vs. longer fragments.

Naked DNA is very rapidly degraded in the blood circulation as the half-life of intact DNA without a double-strand break has been estimated to be less than a minute.^32^ Consequently, only cfDNA protected by stable structures can be detected in the bloodstream. Nuclear cfDNA fragmentation results from mapping locations of the chromatin organization along the genome, which protect/packed DNA with mononucleosomes as the lower unit. At least two key DNA/protein complexes enabling DNA protection from blood nucleases may be considered: DNA wrapped around a histone octamer or DNA bound to transcription factor (TF). Since linker DNA between nucleosomes is vulnerable to digestion, lengths corresponding to one nucleosomal subunit appear to be the most prevalent and conserved size with di- and tri-nucleosomal lengths showing much lower proportions.^16^ Stable nucleosome associated structures may be 192 bp (mononucleosome plus linker), 165 bp (trimmed mononucleosome), or 147 bp (core particle: nucleosome excluding the DNA connected to the peripheral histone H1, which adds ~20 bp; Fig. 3). Trimmed mononucleosome cfDNA-associated structures (165 bp) appear to be preferentially protected as shown by its prevalence in the cfDNA size profile. Note, as already observed by Chan et al.^19^ our data indicate that the fraction cfDNA fragments over the size of DNA wrapped in a mononucleosome as determined by Q-PCR as well as by SSP-S or DSP-S is very minor ( < 1.8% and < 6%, respectively). Whereas size distribution analysis by Q-PCR is not limited to DNA size over ~40 bp, size profile analysis through DSP- or SSP-S, as performed here, is limited to cfDNA of fragments under ~1000 bp and thus precludes examination of cfDNA of higher molecular weight. Nevertheless, cfDNA quantitations were similar when using Q-PCR and SSP-S, suggesting that high molecular weight (over 350 bp) cfDNA is a minor component (~2%) in terms of genome equivalent copy number in cancer patients (Fig. 3). Altogether, this suggest that presence of DNA circulating within di- or oligonucleosomes is minor and that high molecular weight DNA poorly circulate in cancer patients’ blood. Note, this is observed when stringent protocol for the pre-analytical conditions are used. We may postulate that the significant presence of high molecular weight in a cancer patient cfDNA extract could indicate a possible contamination of genomic DNA from lysed blood cells and may be a pre-analytical parameter to assess as quality control of the cfDNA extract.

The presence of cfDNA fragments lower than 100 bp may be explained by various hypotheses. First, degradation at both linker extremities of pieces of DNA protected from TFs previously bound to linker DNA between two nucleosomes (with length ranging between 20 and 90 bp and varying among different species, or tissues; Fig. 3) may release protected short double-stranded fragments into the blood circulation. Second, and more likely, DNA double-stranded or single-stranded breaks may occur in bloodstream inside or outside both types of DNA/protein complexes, inside or outside cells. Following DNA denaturation during PCR or SSP, the resulting single strands may be of varying size. Since we clearly observed the detection of polymerized short double-stranded DNA and sequencing of short sequences from SSP, it is reasonable to assume that the possible sources of detected short ssDNA fragments include both short double-stranded cfDNA (<145 bp) or nicked double-standed cfDNA of higher size. The ~10nt periodicity, within the 41–166nt range, observed with using SSP-S demonstrated the presence of nucleosome-derived degradation since this pattern has been attributed to the internal nucleosome cleavage of accessible nucleotides that lie further from the surface of the histone core at each helical turn as DNA wraps around the core.^15,33^ Observation of periodicity lower than 145 bp down to 81 bp by DSP-S might reveal the presence in blood of short double-stranded DNA associated to nucleosomes. Calculation of the number of reads cannot provide a true estimation of the percentage of nicked intranucleosomal cfDNA. However, since both SSP-S and DSP-S show the same peak at 166 bp we could consider that, at this size, a fraction of cfDNA molecule fragments, at least in one strand, are free of nicks, as illustrated in Fig. 3. DSP-S analysis showed that cfDNAs are principally associated with histones and that the lowest dsDNA fragment length is approximately 80 bp. SSP-S analysis, on the other hand, showed that the detected single-strand cfDNA fragments below the size covered in a mononucleosomal core (<145 nt) were initially mostly associated with histones. This suggests that there is at most only a minor fraction of short histone-free fragments. Since histone association implies dsDNA secondary structure, our data suggest that there is negligible single-stranded DNA circulating in blood.

Note, our previous observations by AFM analysis support the existence of short ds cfDNA structures as a significant proportion of cfDNA from cancer patients was ranging between 100 and 145 bp.^8^ In addition, previous studies of DNase I cleavage patterns identified two dominant classes of fragments: longer fragments associated with cleavage between nucleosomes, and shorter fragments associated with cleavage adjacent to TF-binding sites.^34^ By generating maps of genome-wide in vivo nucleosome occupancy, we previously found that short cfDNA fragments (35–80 bp) harbor footprints of TFs.^27^ Although additional observations from the literature are needed to estimate the proportion or significance of TF-associated cfDNA, higher scrutiny of those short cfDNA fragments might provide a new diagnostic potential based on TF presence. Note, the use of gel electrophoresis assay for cfDNA sizing, which is only based on detecting dsDNA never showed cfDNA fragment length peaking below 180 bp.^1^

All those various reasons concur to the significant discrepancy found in size profile, and that cfDNA structures are of high diversity spanning from tightly packed long dsDNA, mononucleosomes or oligonucleosomes, heminucleosome formation, short-sized TF-binding dsDNA, long-sized and short-sized DNA-associated microparticles, short-sized lipoproteonucleic complexes, and cell or cell-part association. These structures would be all subject to endonuclease and exonuclease degradation as soon as they are released from cells into the blood circulation. Our data showed that nucleosomal structures are one of the least degradable cfDNA structures with, to a lesser extent, TF-associated cfDNA. Apoptosis might appear as the main source of cfDNA; however, short-sized nucleosomal structures could also be the results of the progressive nuclease degradation of higher-sized cfDNA originating from necrosis, phagocytosis, micro-particle-containing DNA, or active release from lymphocytes.^1^ CfDNA structure diversity therefore results from different biological phenomena: various cellular mechanisms of release, dynamic nucleic or proteic degradation in the circulation, and potential association with blood constituents.

We recently demonstrated that deep-sequencing cfDNA for mapping genome-wide in vivo nucleosome occupancy may reveal its tissues of origin.^27^ The many structures and mechanical origins have shown that cfDNA is a complex entity. Sizing following cfDNA extraction cannot fully account for characterizing their structures. Nevertheless, we may hypothesize that the level of fragmentation vary upon cfDNA origins (mitochondrial, nuclear, tumor or healthy cells, lymphocytes, tumor microenvironment cells, metastatic cells, etc.) and information on sizing may be key in accurately detecting and quantifying cfDNA. In the light of this assumption, optimal detection and discrimination of cell-free DNA collected from other body constituents may rely on sizes specific to their origin.

This study shows that much higher cfDNA copies may be readily recovered by selecting/targeting short single-strand fragments, consequently providing higher sensitivity when detecting genetic or epigenetic alterations when testing cancer patient plasma. Based on our initial observation on cfDNA size distribution^18^ and the necessity of targeting short DNA sequences (50–80 bp) for optimal detection by Q-PCR, this strategy was first taken into consideration to an allele specific with blocker Q-PCR method (IntPlex), which demonstrated very high sensitivity,^35,36^ and afterwards when accordingly setting other PCR-based methods, such as single locus Q-PCR,^22^ Beaming,^21^ or dPCR,^23^ or by sequencing and selective amplification.^37,38^ Since cfDNA fragments, and especially mutant cfDNA in cancer patient, may be poorly represented in blood, optimal recovery of cfDNA is required for its analysis. Several reports have showed that SSP-S appears better suited than conventional DSP-S for obtaining an optimal analytical signal. Alternatively, Moser et al.^39^ did not observe a preferential enrichment of circulating DNA.^39^ Thus, it is still debatable as to whether or not SSP will definitively improve the quantification performance, and whether a shift towards SSP-S for analyzing cfDNA in a clinical practice is warranted.

As well as improving cfDNA recovery for optimal detection, knowledge on sizing may also enable subject stratification. We reported that total cfDNA^18^ as well as mutant cfDNA^20^ of cancer patients is more fragmented than that of healthy individuals or of wild-type cfDNA, respectively, by using a Q-PCR-based method. The presence of more fragmented DNA molecules in cancer patients was further elucidated in another study with the use of sequencing technology based on double-stranded library preparation.^20^

The limitations of this study are detailed in the supplementary notes. Briefly, these concern not examining the presence of mitochondria-derived cfDNA, the cancer stages other than stage IV, and potential bias with the extraction procedure. Moreover, the main potential theoretical factors that might contribute to the difference between % SSP-S and % Q-PCR reside in the analytical size window of the methods used here: our ultra-deep-sequencing method spans from ~30 to ~1000 bp and our Q-PCR method over 60 bp. Consequently, our comparative study should be limited to the 60 to ~1000 bp fragment size range. In addition, the study could not determine whether fragment size profiles in cfDNA are associated with tissue types and cancer types as previously reported.^27,40–42^ Furthermore, this study only focused on cancer patient plasma and all resulting observations should not automatically be applied to healthy individual plasma. Although ultra-deep-sequencing analysis showed in previous reports a roughly similar size distribution pattern in healthy and cancer plasma, previous works reported various distinguishing characteristics.^11,18,20,41,42^ We cannot rule out that in-depth scrutiny of size profile may reveal discriminating clear-cut assessment between healthy and cancer patient.

In conclusion, this study confirms the crucial importance of examining the structural features of any analytes circulating in blood, in particular with regards to their association with hetero-compounds. We compared DSP, SSP, and Q-PCR analysis in a blinded study to update and assimilate previous knowledge of cfDNA size profiles. The fragment length distribution of cfDNA, extracted from plasma of cancer patients, was very similar with the SSP-S and Q-PCR methods, which both rely on the analysis of single-stranded DNA as the initial matrix. Both approaches were clearly effective in optimally measuring cfDNA copy number, because a substantial fraction of cfDNA found by these methods consisted of short fragments that are not readily detectable by standard DSP protocols. We also observed that most of the detectable cfDNA in blood, as well as most of the shortest cfDNA fragments (down to ~40 nt), have footprint of a nucleosome, which appears the most stabilizing structure for DNA in the circulation. We conclude that cellular DNAs, initially packaged in chromatin, are released by different biological phenomena in the extracellular compartment in various structures undergoing degradation down to nucleosomes or to a lesser extent TF-associated subcomplexes, resulting from continuous dynamic internucleosomal and intranucleosomal nuclease activity. Thus, detectable cfDNA are mostly composed of a complex mixture of highly degraded DNA as regards to their primary, secondary, or tertiary structures. As sensitivity is clearly a limitation of cfDNA applications, delineating the structural features of cfDNAs may help adapt optimal analytical approaches to study cancer progression or tumor biology.

## Methods

This research was conducted in accordance with all relevant guidelines and procedures, approved by the Institute of Research in Cancerology, and the INSERM.

### Clinical samples

Blood samples were collected from individuals with stage III and IV cancer (IC *n* = 18) (Table 3): colorectal cancer (*n* = 9), lung cancer (*n* = 6), breast cancer (*n* = 2), and liver cancer (*n* = 1). Samples were obtained from Conversant Bio (*n* = 11, Huntsville, AL, USA) and for the post hoc study from the Cancer Institute of Montpellier (ICM; *n* = 7, Val d’Aurelle, Montpellier, France). All patients signed an informed consent and CRC samples from the ICM were obtained from the study EUDRACT 2016-001490-33. Samples were handled accordingly with a pre-analytical guideline previously established by our group.^43^ The study followed the REMARK reporting guidelines. Written informed consent was obtained from all participants.

**Table 3:** Patient clinical characteristics

	Sample ID	Center	Clinical diagnosis	Stage	Gender	Age	cfDNA yield (ng/ml)
DSP-S	IC58	Plasma Lab	Lung cancer (squamous cell carcinoma)	IV	M	63	8.1
	IC61		Lung cancer (small cell)		M	73	7.2
Blind study (SSP-S VS Q-PCR)	IC10		Lung cancer (adenocarcinoma)		F	65	11.4
	IC32		Lung cancer (small cell)		F	69	9.6
	IC33		Colorectal cancer (adenocarcinoma)		M	65	13.8
	IC34		Breast cancer (invasive/infiltrating lobular carcinoma)		F	62	33.6
	IC15		Lung cancer (small cell)		M	70	22.5
	IC17		Liver cancer (hepatocellular carcinoma)		M	62	39
	IC20		Lung cancer (squamous cell carcinoma)		M	60	21.9
	IC35		Breast cancer (ductal carcinoma in situ*)*		F	76	16.2
	IC37		Colorectal cancer (adenocarcinoma)		F	58	15.9
Post hoc (Q-PCR)	IC101	ICM	Colorectal cancer (adenocarcinoma)	IV	M	72	53.9
	IC102		Colorectal cancer (adenocarcinoma)		F	71	14.2
	IC103		Colorectal cancer (adenocarcinoma)		M	–	11.7
	IC104		Colorectal cancer (adenocarcinoma)	III	M	72	29.2
	IC105		Colorectal cancer (adenocarcinoma)	IV	–	–	22.2
	IC108		Colorectal cancer (adenocarcinoma)		M	52	24
	IC109		Colorectal cancer (adenocarcinoma)		M	71	21

Two patient plasma were subjected to DSP-S; nine patient plasma were examined in blind by Q-PCR and SSP-S; seven patient plasma were subjected to Q-PCR in a post hoc analysis. F female, M male, – not available

### Plasma isolation and cfDNA extraction

All blood samples were collected in 4-ml EDTA tubes and plasma DNA were extracted as described in detail in Supplementary Information Materials and methods.

### Preparation of sequencing libraries

Between 0.5 and 10.0 ng of cfDNA were used as inputs for all libraries. Library amplification for all samples was monitored by real-time PCR to avoid over-amplification, and was typically terminated after 4–6 cycles.

#### Preparation of the double-stranded sequencing library

Conventional, double-stranded sequencing libraries were prepared with the ThruPLEX DNA-seq 48D or ThruPLEX Plasma-seq Kits (Rubicon Genomics), comprising a proprietary series of end-repair, ligation, and amplification reactions.

#### Preparation of the single-stranded sequencing library

Single-stranded sequencing libraries were prepared according to a protocol adapted from Gansauge et al.^26^ with using a double-stranded adapter (SI-13) as described in detail in Supplementary Information Materials and methods.

### Size profile analysis by deep sequencing

All libraries were sequenced on HISeq 2000 or NextSeq 500 instruments (Illumina) as described in detail in Supplementary Information Materials and methods.

### Size profile analysis by Q-PCR

The oligonucleotide primers target DNA sequences of increasing size in human *KRAS* region intron 2 (SI-14). The size of the amplicons was 60, 73, 101, 145, 185, 249, and 300 bp. The reverse primer used was the same for all sizes. Our Q-PCR experiments followed the MIQE guideline.^44^ Q-PCR amplifications and analysis were performed as described in detail in Supplementary Information Materials and methods

### Estimation of average DNA molecule length

The average DNA molecule length was estimated according to the method set by Deagle et al.^29^ for quantifying damage in DNA recovered from highly degraded samples^31^ as described in detail in Supplementary Information Materials and methods.

### Analysis of the amplification of short mutant synthetic DNA fragments by Q-PCR

In order to confirm that targeting short sequences amplify the expected fragment size and that no bias exists in the preferential amplification of larger fragment, we amplified two fragments of mutant synthetic DNA of 61 and 103 bp (SI-12) and performed Q-PCR and agarose gel electrophoresis analysis as described in detail in Supplementary Information Materials and methods.

### Statistical analysis

Statistical analysis was performed using the GraphPad Prism V6.01 software. The Student’s *t* test was used to compare means. A probability of <0.05 was considered to be statistically significant: *p=0.05, **p < 0.01; ***p < 0.001; ****p < 0.0001. The Pearson’s test was used for correlation analysis.

## Electronic supplementary material

Supplementary information

## Data Availability

All data generated or analyzed during this study are included in this published article (and its supplementary information files).

## Electronic supplementary material

**Supplementary information** accompanies the paper on the *npj Genomic Medicine* website (10.1038/s41525-018-0069-0).
