# Supplementary material for: New insights into structural features and optimal detection of circulating tumor DNA determined by single-strand DNA analysis
Source: NPJ Genom Med. 2018 Nov 23;3:31. doi: 10.1038/s41525-018-0069-0 (PMC6251887; doi:10.1038/s41525-018-0069-0)
Supplement: Supplementary file 1 — Supplementary information [file 41525_2018_69_MOESM1_ESM.pdf]

Supplementary Information

Study limitations

This study shows some limitations. First, it does not take into consideration mitochondria derived cfDNA in accordance to their growing interest in respect to inflammation and transplant areas of research[1,2]. A few works reported that mitochondria cfDNA do not show a nucleosomal pattern in the plasma of cancer patients and pregnant women and did consistently show a size distribution much shorter (30-115 bp range) than nuclear cfDNA[1,3]. Obviously, mitochondria cfDNA examination would benefit from use of SSP-S[1]. Second, one might think that data obtained here might be depending upon cancer progression stage or type of cancer. Studies with higher number of patient plasma samples from the same malignancy are warranted. Third, discriminating healthy vs cancer patients based on differential fragmentation should be investigated with regards to screening power in light of the initial observation we made with using DNA integrity index[4]. Lastly, we analyzed in this study cfDNA extracts from the mostly used extraction protocol in the literature we previously described[5] and adapted from *Chiu et al*[6]. However, we cannot eliminate the notion that plasma isolation and extraction procedure would affect size profiling. More investigation is needed to evaluate its influence and its potential use it in targeting specific cfDNA fractions.

References:

1. Burnham, P. *et al.* Single-stranded DNA library preparation uncovers the origin and diversity of ultrashort cell-free DNA in plasma. *Scientific Reports* **6,** 27859 (2016).
2. Knez, J. *et al.* Peripheral blood mitochondrial DNA content in relation to circulating metabolites and inflammatory markers: A population study. *PLOS ONE* **12,** e0181036 (2017).
3. Yu, S. C. Y. *et al.* Noninvasive Prenatal Molecular Karyotyping from Maternal Plasma. *PLoS ONE* **8,** e60968 (2013).

4. Mouliere, F. *et al.* High Fragmentation Characterizes Tumour-Derived Circulating DNA. *PLOS ONE* **6,** e23418 (2011).
5. El Messaoudi, S., Rolet, F., Mouliere, F. & Thierry, A. R. Circulating cell free DNA: Preanalytical considerations. *Clinica Chimica Acta* **424,** 222–230 (2013).
6. Chiu, R. W. K. *et al.* Quantitative Analysis of Circulating Mitochondrial DNA in Plasma. *Clinical Chemistry* **49,** 719–726 (2003).
7. Mouliere, F. *et al.* Circulating Cell-Free DNA from Colorectal Cancer Patients May Reveal High KRAS or BRAF Mutation Load. *Translational Oncology* **6,** 319–328 (2013).
8. Gansauge, M.-T. & Meyer, M. Single-stranded DNA library preparation for the sequencing of ancient or damaged DNA. *Nature Protocols* **8,** 737–748 (2013).
9. Bustin, S. A. *et al.* The MIQE guidelines: minimum information for publication of quantitative real-time PCR experiments. *Clinical chemistry* **55,** 611–22 (2009).
10. Jiang, P. & Lo, Y. M. D. The Long and Short of Circulating Cell-Free DNA and the Ins and Outs of Molecular Diagnostics. *Trends in genetics : TIG* **32,** 360–371 (2016).
11. Deagle, B. E., Eveson, J. P. & Jarman, S. N. Quantification of damage in DNA recovered from highly degraded samples – a case study on DNA in faeces. *Frontiers in Zoology* **3,** 11 (2006).
12. Guimaraes, S. *et al.* Owl pellets: a wise DNA source for small mammal genetics. *Journal of Zoology* **298,** 64–74 (2016).
13. Bennett, E. A. *et al.* Library construction for ancient genomics: single strand or double strand? *Biotechniques* **56,** 289–300 (2014).

Materials and methods

Plasma isolation and cfDNA extraction

All samples were collected in 4-ml EDTA tubes. The blood was centrifuged at 1200 g at 4°C for 10 min. The supernatants were isolated in sterile 1.5 ml Eppendorf tubes and centrifuged at 16 000 g at 4°C for 10 min. Afterwards, the plasma was either immediately handled for DNA extraction or stored at -20°C. CfDNA was extracted from 1 ml of plasma using the QiaAMP Circulating Nucleic Acids Kit (Qiagen) according to the manufacturer's protocol for Conversant Bio samples, QIAmp DNA Mini Blood kit (Qiagen) according to the ''Blood and body fluid protocol'' and our detailed protocol[7] for ICM samples. DNA extracts were kept at -20°C until used. DNA yield was quantified with a Qubit Fluorometer (Invitrogen).

Preparation of the single-stranded sequencing library

Single-stranded sequencing libraries were prepared according to a protocol adapted from *Gansauge et al*[8]. Briefly, a double-stranded adapter (SI-13), Adapter2, was prepared by combining 4.5 µl TE (pH 8), 0.5 µl 1M NaCl, 10 µl 500 uM oligo Adapter2.1, and 10 µl 500 uM oligo Adapter2.2, incubating at 95°C for 10 seconds, and ramping to 14°C at a rate of 0.1°C/second. Purified cfDNA fragments were dephosphorylated by combining 2X CircLigase II buffer (Epicentre), 5 mM $MnCl_2$, and 1U FastAP (Thermo Fisher) with 0.5-10 ng cfDNA fragments in 20 µl reaction volume and incubating at 37°C for 30 minutes. Fragments were then denatured by heating to 95°C for 3 minutes and were immediately transferred to an ice bath. The reaction was supplemented with 5 pmol biotin-conjugated adapter oligo CL78, 20% PEG-6000 (w/v), and 200U CircLigase II (Epicentre) for a total volume of 40 µl, and incubated overnight with rotation at 60°C, heated to 95°C for 3 minutes, and placed in an ice bath. For each sample, 20 µl MyOne C1 beads (Life Technologies) were washed twice in bead-binding buffer (BBB) (10 mM Tris-HCl [pH 8], 1M NaCl, 1 mM EDTA [pH 8], 0.05% Tween-20, and 0.5% SDS), and resuspended in 250 µl BBB. Adapter-ligated fragments were bound to the beads by rotating for 60 minutes at room temperature. The beads were collected on a magnetic rack and the supernatant was discarded. The beads were washed once with 500 µl wash buffer A (WBA) (10 mM Tris-HCl [pH 8], 1 mM EDTA [pH 8], 0.05% Tween-20, 100 mM NaCl, 0.5% SDS) and once with 500 µl wash buffer B (WBB) (10 mM Tris-HCl [pH 8], 1 mM EDTA [pH 8], 0.05% Tween-20, 100 mM NaCl). The beads were combined with 1X Isothermal Amplification Buffer (NEB), 2.5 uM oligo CL9, 250 uM (each) dNTPs, and 24U Bst 2.0 DNA Polymerase (NEB) in a

reaction volume of 50 µl, incubated with gentle shaking by ramping temperature from 15°C to 37°C at 1°C/minute, and held at 37°C for 10 minutes. After collection on a magnetic rack, the beads were washed once with 200 µl WBA, resuspended in 200 µl of stringency wash buffer (SWB) (0.1X SSC, 0.1% SDS), and incubated at 45°C for 3 minutes. The beads were collected again and washed once with 200 µl WBB. The beads were then combined with 1X CutSmart Buffer (NEB), 0.025% Tween-20, 100 uM (each) dNTPs, and 5U T4 DNA Polymerase (NEB), and incubated with gentle shaking for 30 minutes at room temperature. The beads were washed once with each of WBA, SWB, and WBB as described above. The beads were then mixed with 1X CutSmart Buffer (NEB), 5% PEG-6000, 0.025% Tween- 20, 2 uM double-stranded Adapter2, 1 mM ATP, and 10U T4 DNA Ligase (NEB), and incubated with gentle shaking for 2 hours at room temperature. The beads were washed once with each of WBA, SWB, and WBB as described above, and resuspended in 25 µl TET buffer (10 mM Tris-HCl [pH 8], 1 mM EDTA [pH 8], 0.05% Tween-20). Second strands were eluted from the beads by heating to 95°C, collecting them on a magnetic rack, and transferring the supernatant to a new tube. Library amplification was monitored by real-time PCR, requiring an average of 4-6 cycles per library.

Size profile analysis by deep sequencing

All libraries were sequenced on HISeq 2000 or NextSeq 500 instruments (Illumina). Barcoded single-end reads (SR) and the individual barcoded paired-end reads (PE) were aligned to the human reference genome (GRCH37, 1000 Genomes phase 2 technical reference, ftp://ftp.1000genomes.ebi.ac.uk/vol1/ftp/technical/reference/phase2_reference_assembly_sequence/) using the ALN algorithm in BWA v0.7.10. PE reads were further processed with BWA SAMPE to resolve ambiguous placement of read pairs or to rescue missing alignments by a more sensitive alignment step around the location of one placed read end. Aligned SR and PE data were stored in BAM format using the samtools API. BAM files for each sample were merged across lanes and sequencing runs. Since SSP-S provides size profile based on detection of ssDNA fragment, length unit was consequently number of nucleotide.

Size profile analysis by Q-PCR

The oligonucleotides primers target DNA sequences of increasing size in human *KRAS* region intron 2 (SI-14). The size of the amplicons was 60 bp, 73 bp, 101 bp, 145 bp, 185 bp, 249 bp, and 300 bp. The reverse primer used was the same for all sizes. Our Q-PCR experiments followed the MIQE guideline[9]. Q-PCR amplifications were performed at least in duplicate in a 25 µl reaction volume on a CFX96 instrument using the CFX manager software 3.0 (Bio-Rad). Each PCR reaction mixture was composed of 12.5 µl PCR mix (Bio-Rad Super mix SYBR Green), 2.5 µl of each amplification primer (0.3 pmol/µl, final concentration), 2.5 µl PCR-analyzed water, and 5 µl DNA extract. Thermal cycling consisted of three repeated steps: a 3-min Hot-start Polymerase activation denaturation step at 95°C followed by 40 repeated cycles at 95°C for 10 s and then at 60°C for 30 s. Melting curves were obtained by increasing the temperature from 55°C to 90°C with a plate reading every 0.2°C. As calibrators for quantification, serial dilutions of genomic DNA from the DIFI cell line were used. Sample concentrations were extrapolated from this standard curve. Negative controls were used in duplicate for each experiment. The efficiency of these primers was assessed using as the reference a human genomic DNA purchased from Promega. This DNA was quantified by Q-PCR using the different primer systems showed a Gaussian distribution with maximum amplification with primers targeting the 145 nt sequence (SI-15). Each concentration obtained for each size was normalized based on the concentration observed using the primers targeting the 145 nt sequence. The DNA concentration corresponds to the number of amplicons obtained for each targeted size. Size range fractions were calculated by subtracting the amount obtained with the larger amplicon from the amount obtained with the shorter amplicon divided by the highest amplicon amount (60 bp minus 73 bp divided by 60 bp, 60 bp minus 101 bp divided by 60 bp). Since Q-PCR provides a size profile based on detection of ssDNA fragments, the length unit is consequently number of nucleotide. The fraction corresponding to cfDNA fragments higher than 300 nt or 249 nt corresponds to the amount obtained when targeting sequence of 300 nt or 249 nt. A schematic representation is presented in Fig. 1*C* adapted from *Jiang et al.*[10]. The size distribution was also represented in three fractions: HF, Highly fragmented ssDNA (<145 nt); MF, Mononucleosomal ssDNA fragments (145-249 nt); and WF, weakly fragmented ssDNA (> 249 nt). The Q-PCR analyses were carried out blinded in respect to the NGS sequencing data.

Estimation of average DNA molecule length

The average DNA molecule length was estimated according to the method set by Deagle[11] for quantifying damage in DNA recovered from highly degraded samples[12]. It is based on the rationale that DNA degradation occurs randomly and follows first-order kinetics. The approach uses Q-PCR to measure the amount of amplifiable target DNA for fragments of various sizes within a single sample. If DNA damage occurs according to a random Poisson process at a rate of λ, then there is expected to be an exponential decline in the amount of amplifiable product with increasing product size, and the rate of decline is sharper for higher values of λ. The resulting size distribution of undamaged molecules (x) is defined by an exponential distribution with parameter λ: $Ax = N \exp(-\lambda x)$ where Ax is the number of amplifiable copies of size x and N is the total number of DNA copies. Thus, data are log-transformed and a linear model is fitted in order to evaluate the probability of a nucleotide being damaged (λ). We accordingly plotted the quantity of DNA fragments obtained with 60, 73, 101, 145, and 185 nt obtained from each samples against the amplicon length (bp) and calculated the average molecule length ($1/\lambda$).

Analysis of the amplification of short mutant synthetic DNA fragments by Q-PCR

In order to confirm that targeting short sequences amplify the expected fragment size and that no bias exists in the preferential amplification of larger fragment, we designed two fragments of mutant synthetic DNA and they were synthetized by Eurofins Genomics GeneStrands. These fragments contain the G12V mutated *KRAS* exon 2 gene sequence. The fragment lengths are 61 bp and 103 bp, respectively, and they were amplified by Q-PCR using a primers targeting a sequence of 61 bp (SI-14), and the products were analyzed by agarose gel electrophoresis. The amplicon size was 61 bp corresponding to the mutated DNA sequence. The Q-PCR amplification conditions were the same except that the water in the SYBR mix was replaced by an oligoblocker, which targets the non-mutated DNA sequence. As calibrators for quantification, serial dilutions of genomic DNA from the SW620 cell line were used. A positive control of the mutation from the SW620 cell line was amplified. A negative control of the mutation from the DIFI cell line was amplified. Agarose gel was performed with 2.75% ultrapure agarose 1000 (Invitrogen). We prepared DNA samples with 6X DNA Loading dye, 1 volume for 5 volume of DNA. For the PCR products, we loaded 20 µl of each sample and for the synthetic DNA fragments 30 µl (54 ng of DNA).

Supplementary Information (Figures and Tables)

SI-1: Study work plan. The same set of cfDNA extracts from cancer patients were analyzed in blind by both deep sequencing from SSP and DSP libraries and Q-PCR (right panel). Schematic diagram of SSP and DSP library preparation adapted from Bennett et al[13] (left panel). Analytical method of fractional fragment size distribution of cfDNA of cancer patients (right panel). **A,** Nested PCR primer systems used to analyze the size distribution profile. **B,** Concentration of DNA corresponding to each amplicon size. **C,** Illustration of the fractional fragment size profile calculation.

SI-2: Zoom of the size profile between 280 to 360 nt or bp obtained from the cancer samples by SSP-S (full lines) and DSP-S (dotted lines), respectively. A weak DNA fragment sub-population (<3% of total reads in that range) peaking at 307 and 308 bp (IC58 and IC61, respectively) which may correspond to the size of DNA contained in a di-nucleosome, is detectable by DSP-S. No sharp increase of reads, as obtained by SSP-S, is observable within that range whereas a more than two-fold increase is detected from 280 bp to the peaking size (307 and 308 bp in IC58 and IC61, respectively) by DSP-S. Both peaks correspond to 1.8 % of the reads measured at the peaking size (166 bp for both samples analyzed by DSP-S). Percent of fragments are calculated from the total reads obtained from 30 to 1000 nt or bp.

SI-3: Fractional fragment size profile of illustrative cfDNA originating from cancer patient by Q-PCR method (blinded comparative study).

SI-4: **Direct comparison of the fractional fragment size distribution of cfDNA from cancer patients as determined by deep sequencing with SSP library (a) and by Q-PCR method (b).** HF, Highly fragmented DNA (<145 bp); MF + WF (>145 bp).

SI-5: Fractional fragment size distribution by SSP-S of cfDNA from cancer patients. HF, Highly fragmented DNA (<145 nt); MF, Mono-nucleosome Fragmented DNA (145-249 nt); WF, Weakly Fragmented DNA (> 249 nt).

SI-6: **Direct comparison of the fractional fragment size distribution of cfDNA from cancer patients.** HF, MF and WF size fractions from IC35, IC17 and IC34 patient plasma (a) and HF and MF+WF size fractions from IC15, IC20, IC32, IC10, IC35, IC34, IC33, IC37, IC17 (b) determined by deep sequencing with SSP library (blue) and Q-PCR analysis (orange).

SI-7: Direct comparison of the fractional fragment size distribution of cfDNA from cancer patients as determined by SSP and Q-PCR method. a, HF, Highly fragmented DNA (<145 bp); MF, Mononucleosomal DNA fragments (145-249); and WF, weakly fragmented DNA (> 249). **b,** HF, Highly fragmented DNA (<145 bp); MF + WF (> 145).

SI-8: **Combined data from all tested plasma by either Q-PCR or SSP sequencing**. **a,** cfDNA fractional fragment size distribution from cancer patients (n=16) by Q-PCR method (p= 2,7.10-21); **b,** cfDNA fractional fragment size distribution from cancer patient (n=9) by SSP sequencing method (p=7.35.10-9). HF, Highly Fragmented DNA (<145 nt); MF, Mono-nucleosome fragmented DNA (145-249 nt); WF, Weakly Fragmented DNA (> 249 nt); MF + WF (> 145 nt). For both analytical approaches HF fraction is statistically different to the MF + WF fraction when using either Q-PCR or SSP-S (p= 2,7.10-21, and p=7.35.10-9, respectively).

SI-9: **Fractional fragment size distribution (60 to >249bp) of cfDNA originating from cancer patients from Q-PCR analysis.**

SI-10: **Fractional fragment size profile of illustrative cfDNA originating from colorectal cancer patient (added, n=7).**

SI-11: Estimation of average DNA molecule length. The average DNA molecule length was estimated according to the method set by Deagle[11]. A, The resulting size distribution of undamaged molecules (x) is defined by an exponential distribution with parameter λ: $Ax = N \exp(-\lambda x)$ where Ax is the number of amplifiable copies of size x and N is the total number of

DNA copies (Material and Methods online). B, Correlation between values obtained from the average length as determined by Deagle's method and values obtained from Q-PCR fractional analysis.

SI-12: **Q-PCR enables amplification of DNA fragments of the targeted sequence with the same efficiency and the same size.** Agarose gel 2.75% electrophoresis on a white background, G12V mutant synthetic DNA fragments (61 and 103 bp), and the genomic DNA SW620 cell line was amplified by Q-PCR and the products were analyzed by agarose gel electrophoresis. Lane L1 : 50 bp DNA ladder marker; lane Neg C1 = negative control (water + primer targeting the mutation); lane Neg C2 = negative control (DIFI DNA cell line + primer targeting the mutation); lane Pos C1= genomic mutated DNA from SW620 cell line + primers targeting G12V mutation (amplicon length=61 bp); lane 61: 61 bp mutated DNA fragments + primers targeting G12V mutation (amplicon length=61 bp); lane F61: 61 bp mutated DNA synthetic fragments (54 ng) from eurofins; lane 103: 103 bp mutated DNA fragments + primers targeting G12V mutation (amplicon length=61 bp); lane L2= 50 bp DNA ladder marker; lane Pos C2: genomic DNA from SW620 cell line; lane Pos C3: genomic DNA from DIFI cell line.

SI-13: Synthetic oligos used in preparation of single stranded sequencing libraries. Sequences adapted from Gansauge M-T & Meyer M[8]

* : phosphorothioate bond

/5Phos/ : 5' phosphorylation

/ddT/ : dideoxy T

/iSpC3/ : three carbon spacer arm

/3BioTEG/ : 3' Biotin-TEG

SI-14: Characteristics of the selected primers and corresponding amplicons.

SI-15: Quantification from human genomic DNA (Promega) upon Q-PCR targeting sequences of increasing size.

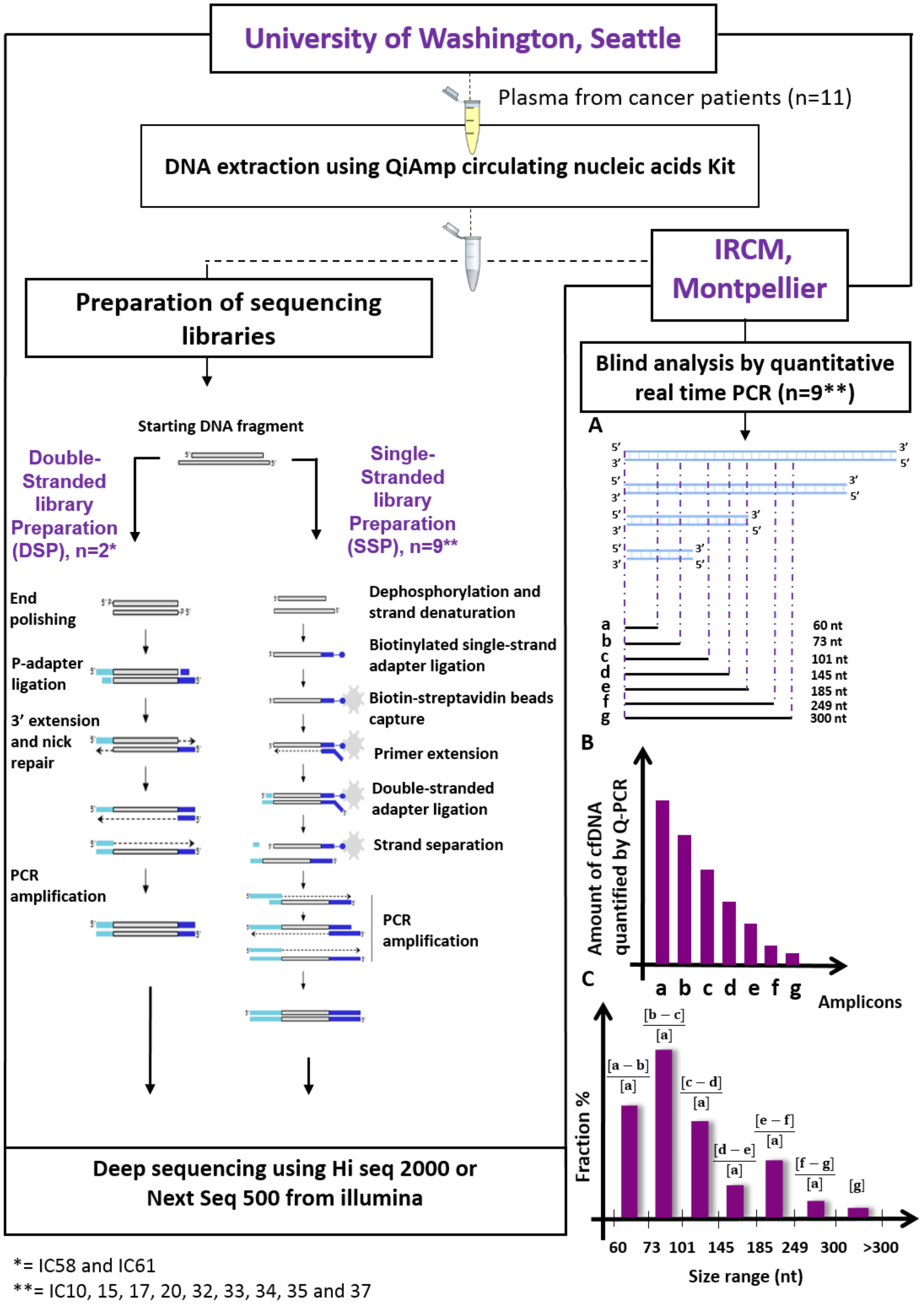

*= IC58 and IC61

**= IC10, 15, 17, 20, 32, 33, 34, 35 and 37

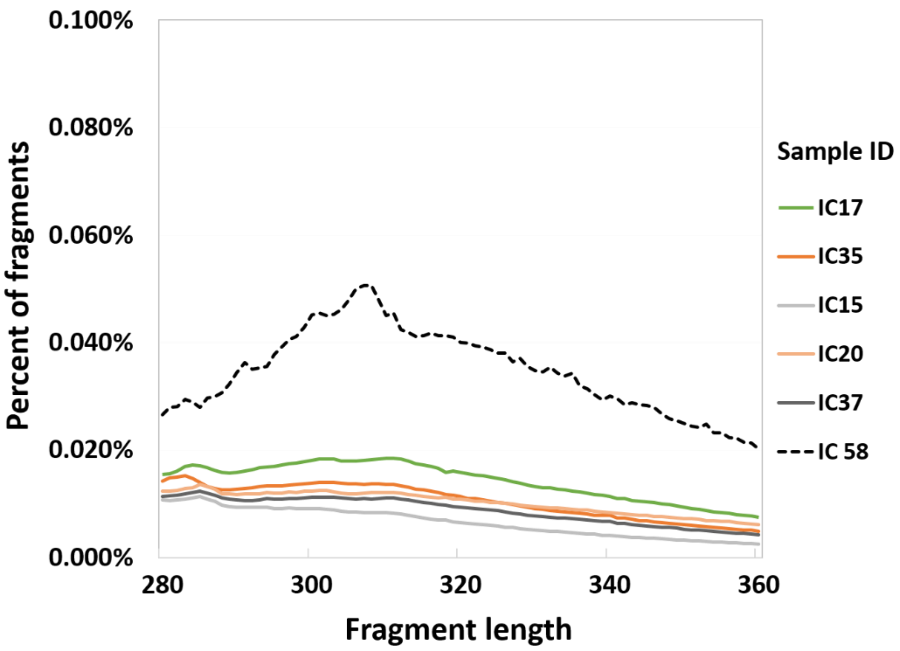
0.100%
0.080%
0.060%
0.040%
0.020%
0.000%
Percent of fragments
280
300
320
340
360
Fragment length
Sample ID
IC17
IC35
IC15
IC20
IC37
IC 58

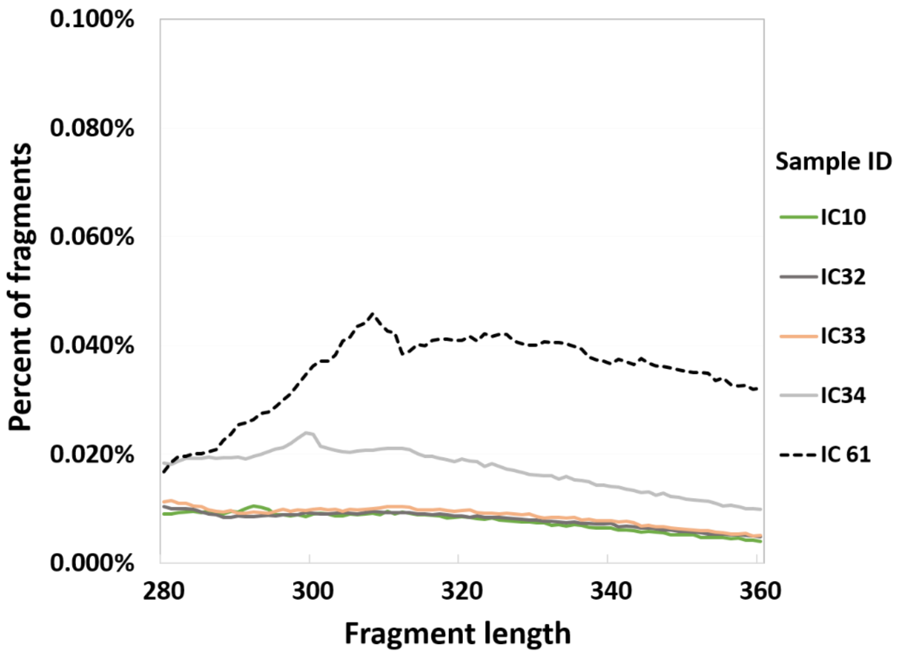
0.100%
0.080%
0.060%
0.040%
0.020%
0.000%
Percent of fragments
280
300
320
340
360
Fragment length
Sample ID
IC10
IC32
IC33
IC34
IC 61

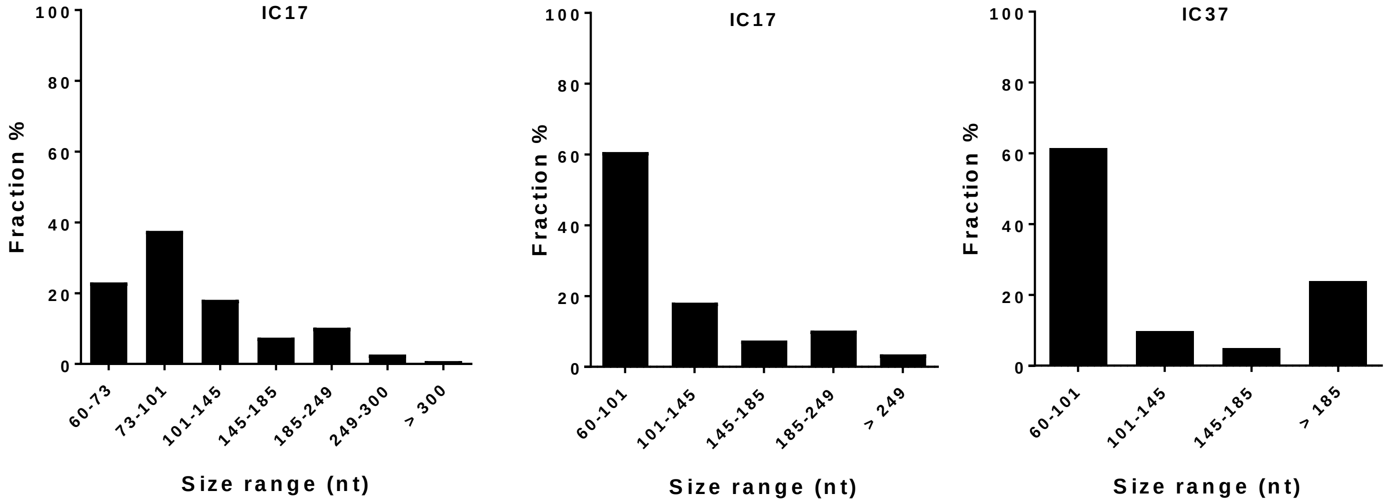
IC17
Fraction %
Size range (nt)
60-73
73-101
101-145
145-185
185-249
249-300
> 300
IC17
60-101
101-145
145-185
185-249
> 249
IC37
60-101
101-145
145-185
> 185

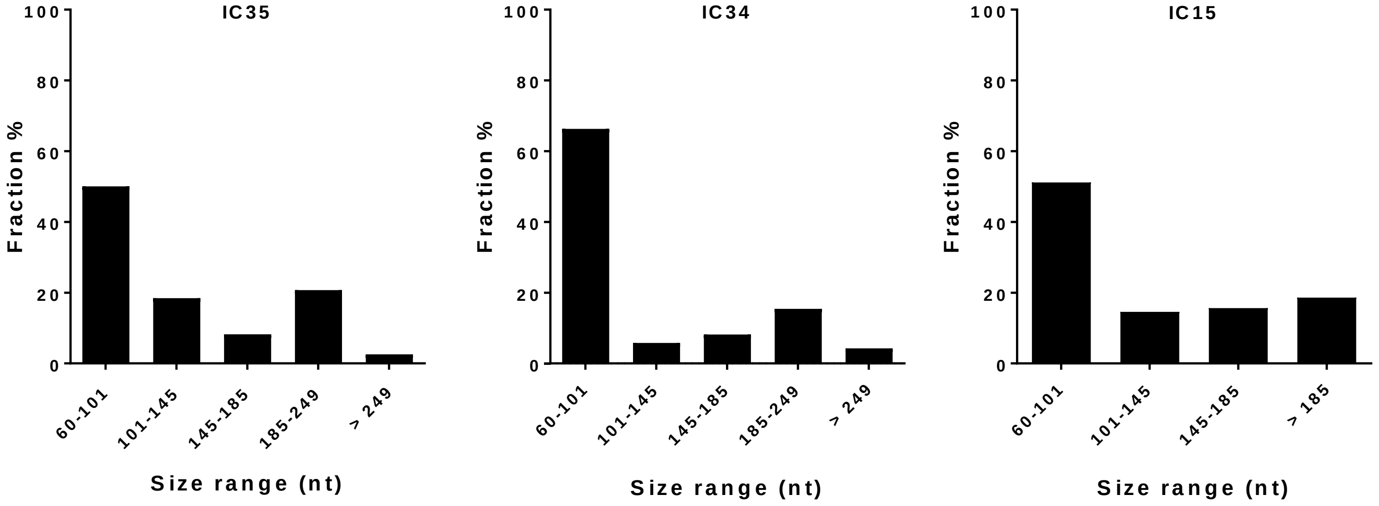
IC35
Fraction %
Size range (nt)
60-101
101-145
145-185
185-249
> 249
IC34
IC15
> 185

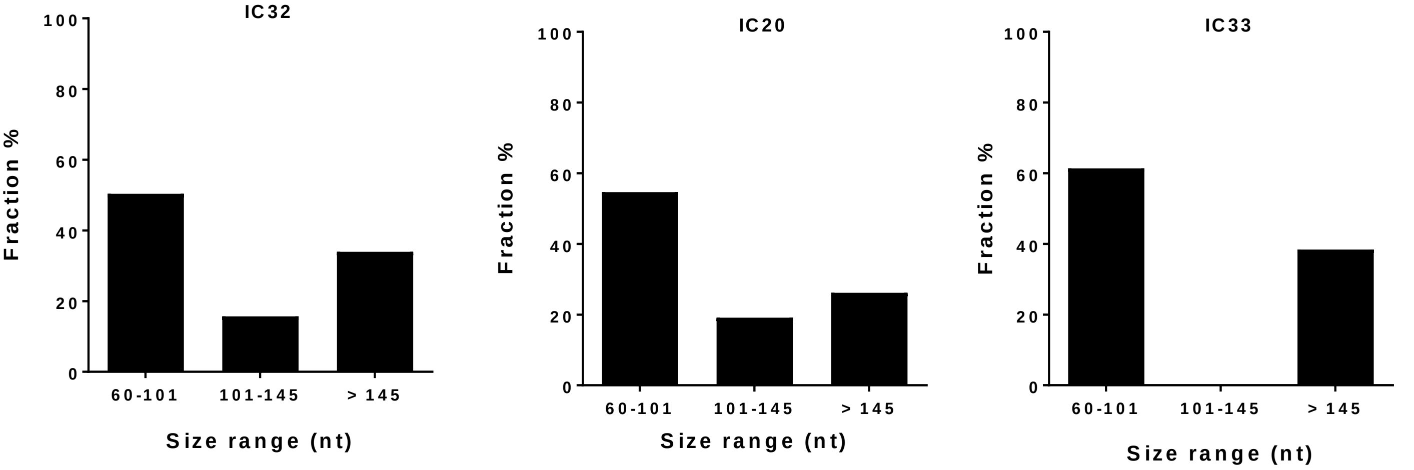
IC32
Fraction %
Size range (nt)
60-101
101-145
> 145
IC20
IC33

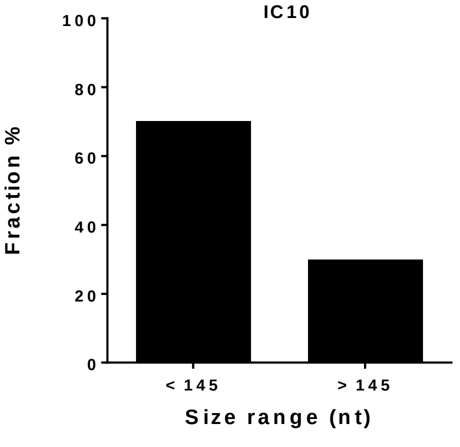
IC10
Fraction %
Size range (nt)
< 145
> 145

a

Origin	Sample ID	Clinical site	Patient sex	Types of cancer	Stage	Fraction	
						HF	MF+WF
Blind study	IC32	PlasmaLab	F	Lung	IV	71,4%	28,6%
	IC10					65,8%	34,2%
	IC15		M			77,0%	23,0%
	IC20					65,0%	35,0%
	IC17		M	Liver		55,4%	44,6%
	IC37		F	Colorectal		70,0%	30,0%
	IC33		M			66,7%	33,3%
	IC35		F	Breast		69,6%	30,4%
	IC34					54,7%	45,3%
**Fraction Mean**						**66,2%**	**33,8%**
**Standard deviation (SD)**						**7,2**	**7,2**

b

Origin	Sample ID	clinical site	Patient sex	Types of cancer	Stage	Fraction	
						HF	MF + WF
Blind study	**IC15**	**PlasmaLab**	M	Lung	IV	65,8%	34,3%
	**IC20**		M			73,8%	26,2%
	**IC10**		F			70,1%	29,9%
	**IC32**		F			66,0%	34,0%
	**IC35**		F	Breast		68,5%	31,5%
	**IC34**		F			72,1%	27,9%
	**IC33**		M	Colorectal		61,6%	38,4%
	**IC37**		F			71,2%	28,8%
	**IC17**		M	Liver		78,8%	21,2%
Post-hoc	**IC101**	**ICM**	M	Colorectal		81,1%	18,9%
	**IC102**		F			72,1%	27,9%
	**IC103**		M			69,6%	30,4%
	**IC104**		-			76,2%	23,8%
	**IC105**		-			69,4%	30,6%
	**IC108**		M			72,7%	27,3%
	**IC109**		M			76,5%	23,5%
**Fraction mean**						**71,6%**	**28,4%**
**Standard deviation (SD)**						**5**	**5**

Sample ID	SSP sequencing		
	HF	MF	WF
IC32	71,4%	27,3%	1,3%
IC10	65,8%	33,0%	1,2%
IC15	77,0%	21,9%	1,1%
IC20	65,0%	33,4%	1,7%
IC17	55,4%	42,5%	2,0%
IC37	70,0%	28,6%	1,4%
IC33	66,7%	31,9%	1,3%
IC35	69,6%	28,8%	1,6%
IC34	54,7%	42,8%	2,5%
**Fraction Mean**	**66,2%**	**32,2%**	**1,6%**
**Standard Deviation (SD)**	**7,2**	**6,8**	**0,4**

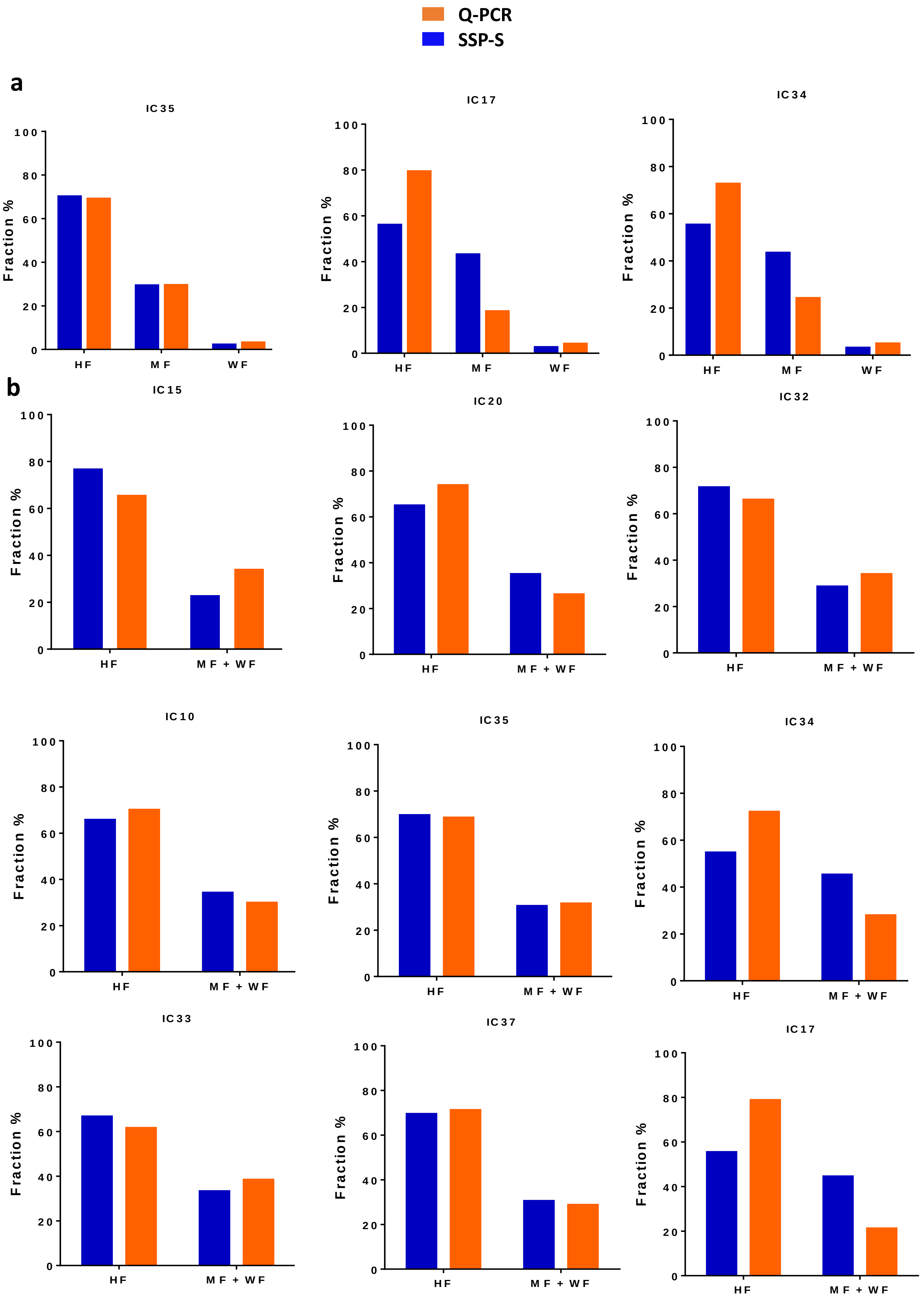

Q-PCR
SSP-S
a
IC35
IC17
IC34
b
IC15
IC20
IC32
IC10
IC35
IC34
IC33
IC37
IC17
Fraction %
100
80
60
40
20
0
HF
MF
WF
MF + WF

a

Origin	Sample ID	Patient sex	Types of cancer	Stage	Fraction					
					HF		MF		WF	
					q-PCR	SSP	q-PCR	SSP	q-PCR	SSP
Blind study	**IC35**	F	Breast	IV	68,5%	69,6%	28,9%	28,8%	2,6%	1,6%
	**IC34**	F			72,1%	54,7%	23,6%	42,8%	4,3%	2,5%
	**IC17**	M	Liver		78,8%	55,4%	17,7%	42,6%	3,5%	2,0%
**Fraction mean**					**73,1%**	**59,9%**	**23,4%**	**38,0%**	**3,5%**	**2,0%**
**Standard deviation (SD)**					**5,2**	**8,4**	**5,6**	**8,0**	**0,9**	**0,5**

b

Origin	Sample ID	Patient sex	Types of cancer	Stage	Fraction			
					HF		MF + WF	
					q-PCR	SSP	q-PCR	SSP
**Blind study**	**IC15**	M	Lung	IV	65,8%	77,0%	34,3%	23,0%
	**IC20**	M			73,8%	65,0%	26,2%	35,0%
	**IC10**	F			70,1%	65,8%	29,9%	34,2%
	**IC32**	F			66,0%	71,4%	34,0%	28,6%
	**IC35**	F	Breast		68,5%	69,6%	31,5%	30,4%
	**IC34**	F			72,1%	54,7%	27,9%	45,3%
	**IC33**	M	Colorectal		61,6%	66,7%	38,4%	33,3%
	**IC37**	F			71,2%	70,0%	28,8%	30,5%
	**IC17**	M	Liver		78,8%	55,4%	21,2%	44,6%
**Fraction mean**					**69,8%**	**66,2%**	**30,2%**	**33,9%**
**Standard deviation (SD)**					**5,1**	**7,2**	**5,1**	**7,2**

a

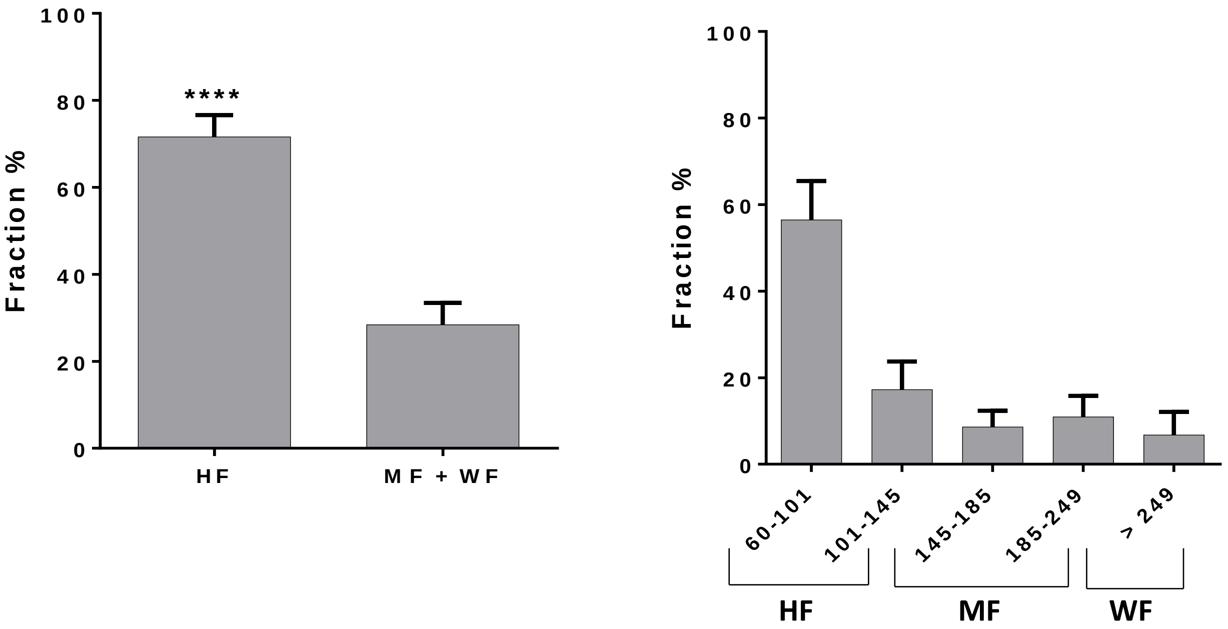

Fraction %
100
80
60
40
20
0
****
HF
MF + WF
Fraction %
100
80
60
40
20
0
60-101
101-145
145-185
185-249
> 249
HF
MF
WF

b

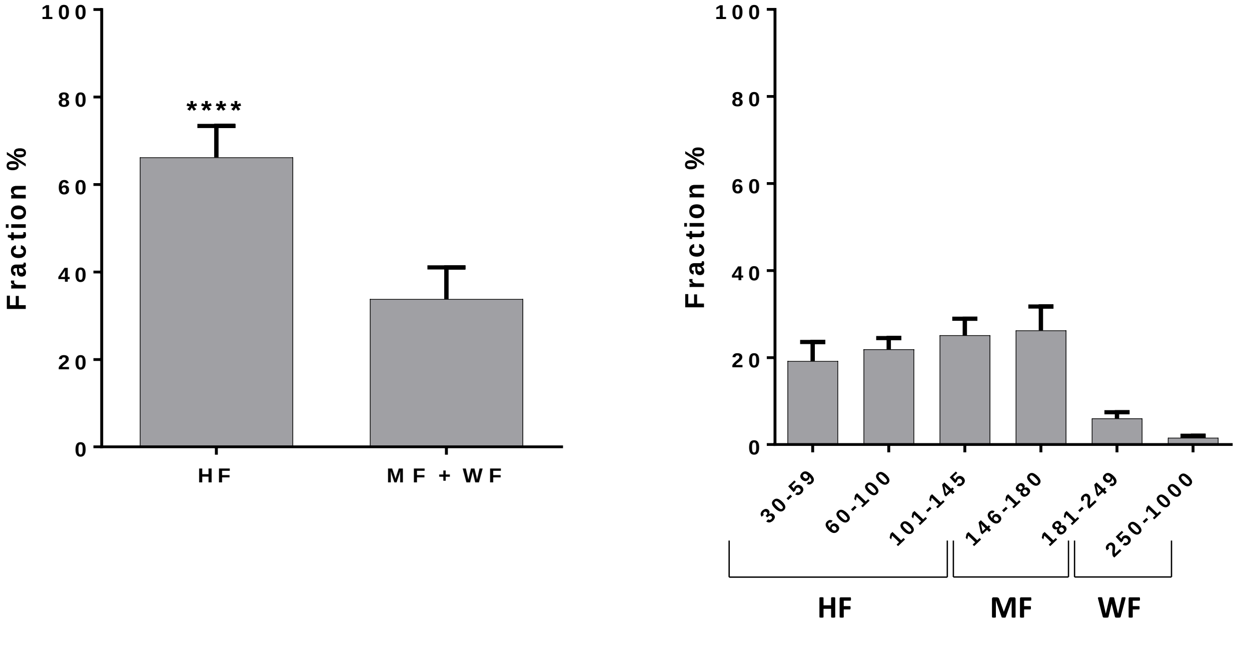

Fraction %
100
80
60
40
20
0
****
HF
MF + WF
Fraction %
100
80
60
40
20
0
30-59
60-100
101-145
146-180
181-249
250-1000
HF
MF
WF

Origin	Sample ID	clinical site	Patient sex	Types of cancer	Stage	cfDNA yield (ng/ml)	Fraction				
							**60-101**	**101-145**	**145-185**	**185-249**	**> 249**
Blind study	**IC35**	**PlasmaLab**	F	Breast	IV	16,2	50,1%	18,4%	8,2%	20,7%	2,6%
	**IC34**		F			33,6	66,3%	5,8%	8,2%	15,5%	4,3%
	**IC17**		M	Liver		39,0	60,7%	18,1%	7,5%	10,3%	3,5%
Post-hoc	**IC101**	**ICM**	-	Colorectal		53,9	70,3%	10,8%	8,8%	6,7%	3,4%
	**IC102**		-			14,2	52,4%	19,7%	8,8%	6,7%	12,5%
	**IC103**		-			11,7	53,1%	16,5%	15,3%	7,6%	7,5%
	**IC104**		-			29,2	58,4%	17,7%	10,1%	12,6%	1,1%
	**IC105**		-			22,2	38,3%	31,1%	12,5%	15,2%	3,0%
	**IC108**		-			24,0	54,5%	18,2%	5,5%	6,8%	15,0%
	**IC109**		-			21,0	60,3%	16,2%	1,5%	7,4%	14,7%
**Fraction mean**							**56,4%**	**17,3%**	**8,6%**	**10,9%**	**6,8%**
**Standard deviation (SD)**							**9,0**	**6,5**	**3,7**	**4,9**	**5,3**

SI-9

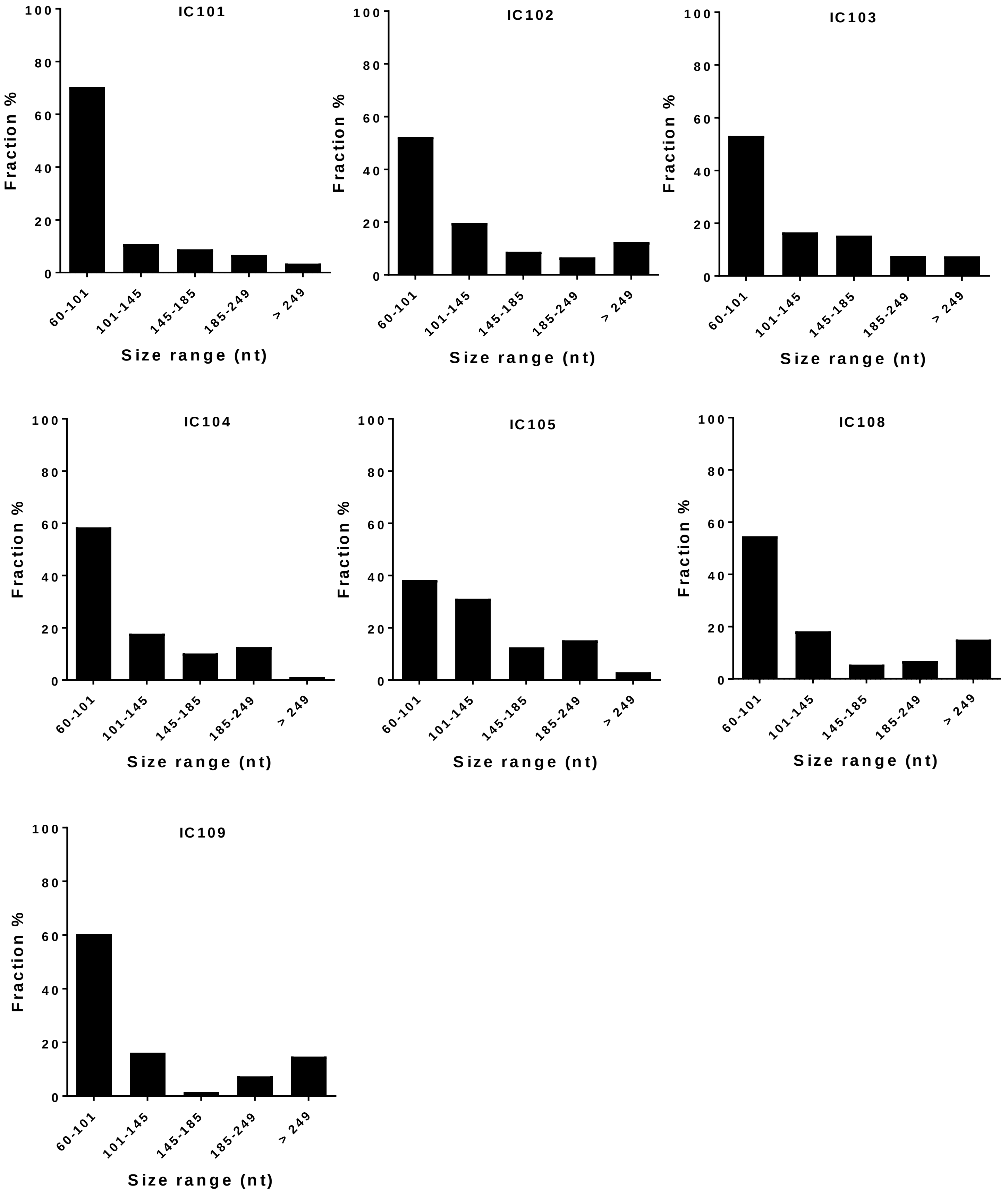
IC101
IC102
IC103
IC104
IC105
IC108
IC109
Fraction %
Size range (nt)
0
20
40
60
80
100
60-101
101-145
145-185
185-249
> 249

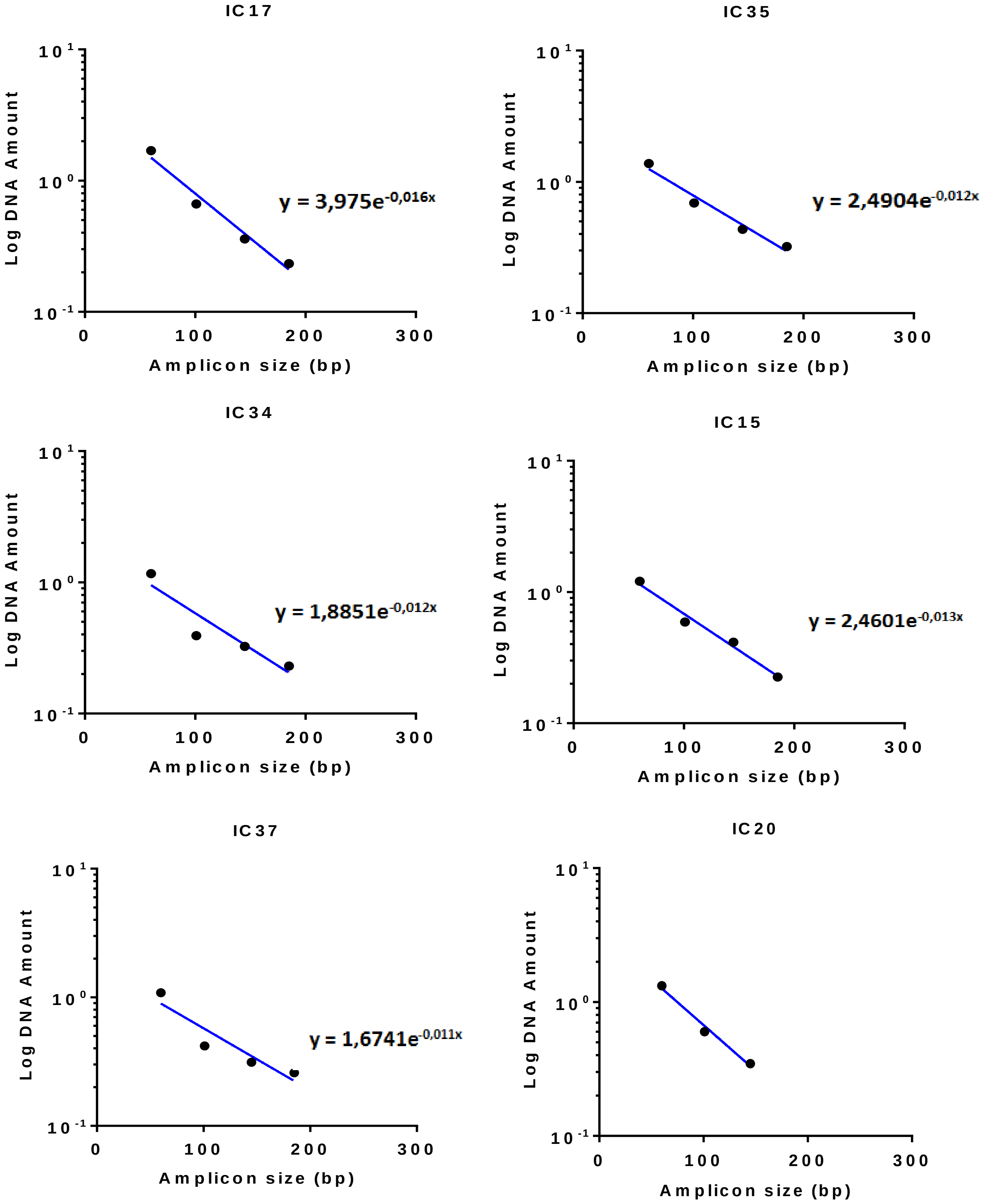

IC17
$y = 3{,}975e^{-0{,}016x}$
IC35
$y = 2{,}4904e^{-0{,}012x}$
IC34
$y = 1{,}8851e^{-0{,}012x}$
IC15
$y = 2{,}4601e^{-0{,}013x}$
IC37
$y = 1{,}6741e^{-0{,}011x}$
IC20
Log DNA Amount
Amplicon size (bp)
10^1
10^0
10^{-1}
0
100
200
300

SI-11 a

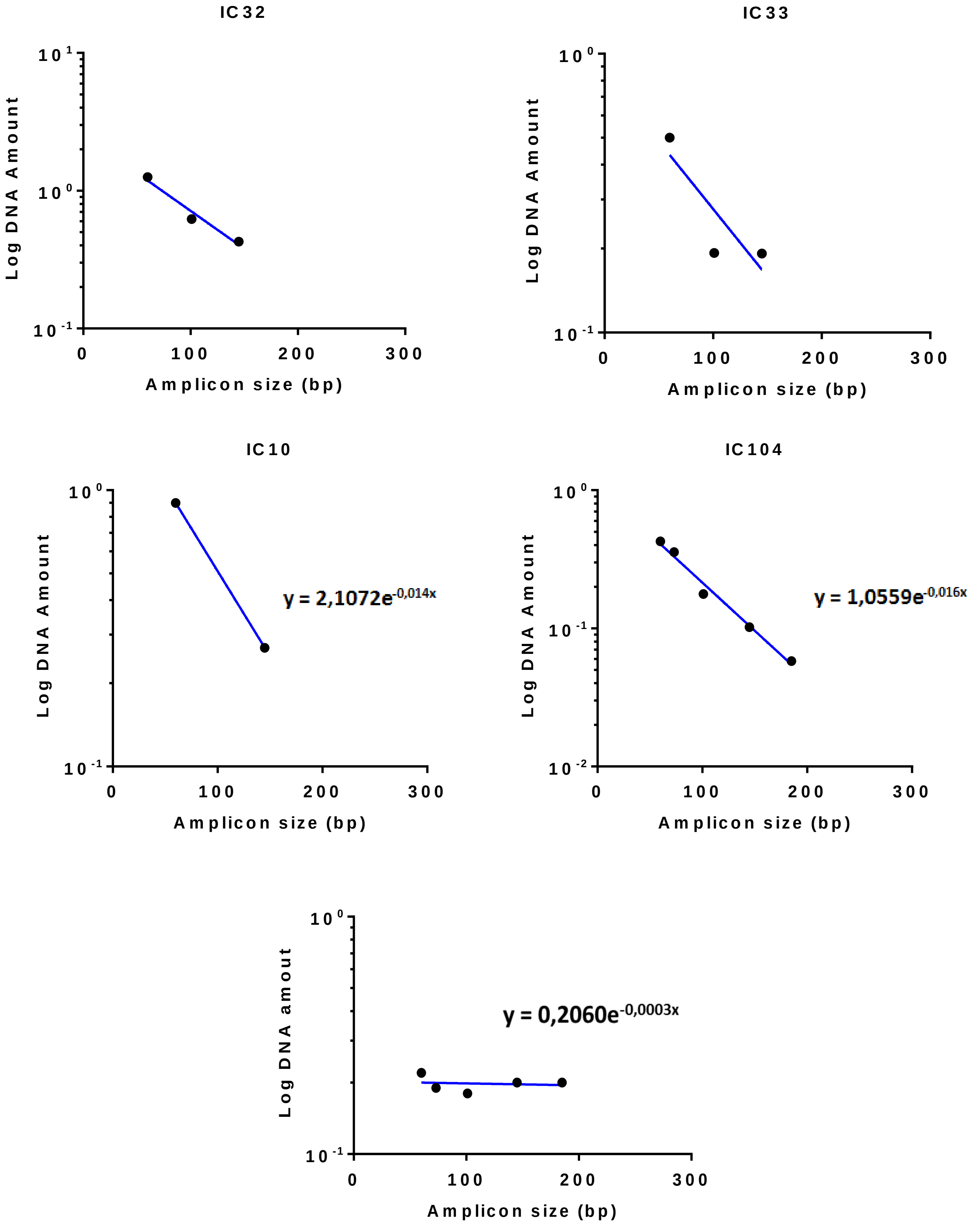
IC32
IC33
IC10
IC104
Log DNA Amount
Log DNA amout
Amplicon size (bp)
y = 2,1072e^-0,014x
y = 1,0559e^-0,016x
y = 0,2060e^-0,0003x

SI-11 a

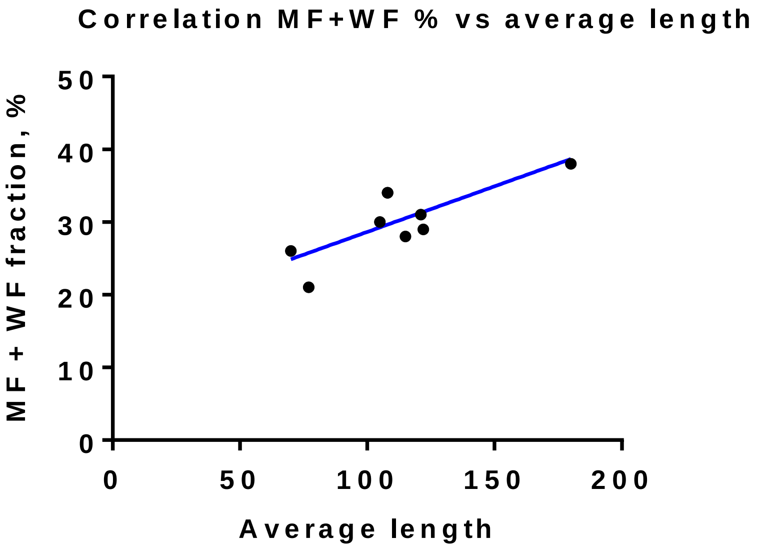
Correlation MF+WF % vs average length
MF + WF fraction, %
Average length
0
10
20
30
40
50
0
50
100
150
200

SI-11 b

Q-PCR amplification using primers that target *KRAS* exon 2 G12V mutation

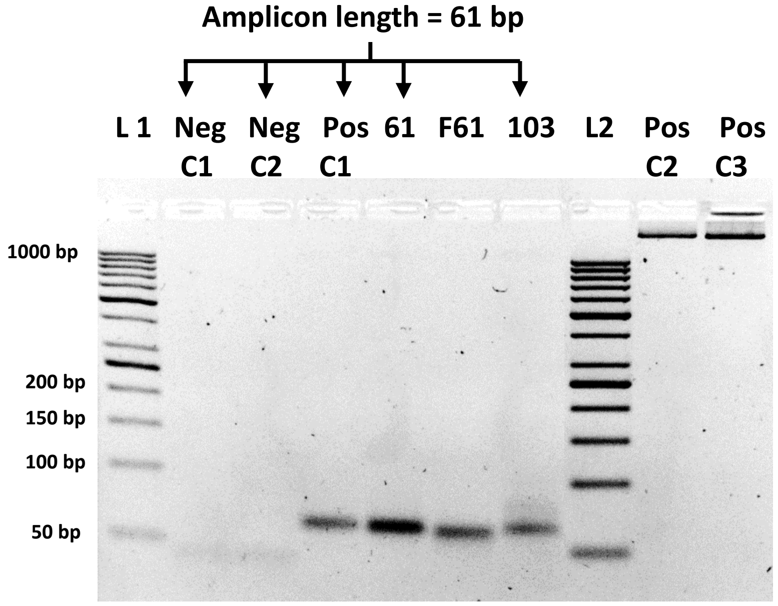

Oligo Name	Sequence (5′-3’)	Notes
**CL9**	GTGACTGGAGTTCAGACGTGTGCTCTTCCGATCT	HPLC purification
**Adapter2.1**	CGACGCTCTTCCGATC/ddT/	HPLC purification
**Adapter2.2**	/5Phos/AGATCGGAAGAGCGTCGTGTAGGGAAAGAG*T*G*T*A	HPLC purification
**CL78**	/5Phos/AGATCGGAAG/iSpC3/iSpC3/iSpC3/iSpC3/iSpC3/iSpC3/ iSpC3/iSpC3/iSpC3/iSpC3/3BioTEG/	Dual HPLC purification

Specie	gene	location	primer name	direction	sequence 5'-3'	Tm (°C)	amplicon size (bp)
**Human**	***KRAS***	Intron 2	Kras 60	Forward	GCCTGTGACCTACAGTGAAAA	57.9	60
			Kras 73	Forward	CCCTACACGTAGAGCCTGTGA	61.8	73
			Kras 101	Forward	GAGATGGTGGAAGAACAGGTG	59.8	101
			Kras 145	Forward	TGGGCTGTGACATTGCTG	60.4	145
			Kras 185	Forward	ATCTGCCTCCGAGTTCCTG	58.8	185
			Kras 249	Forward	TGGAAGAGCATAGGAAAGTGC	57.9	249
			Kras 300	Forward	GGTCCTTTTCCGTGTGTAGG	59.4	300
			Kras 145-300r	Reverse	TGACCAAGCAAAACAGACCA	60.2	-
		Exon 2	Kras A1	Forward	GCCTGCTGAAAATGACTGA	54.5	61
			Kras G12V	Reverse	CTCTTGCCTACGCCAA	51.9	-

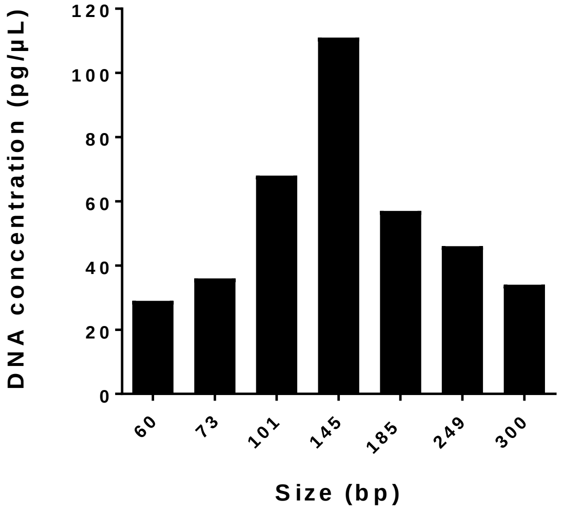

DNA concentration (pg/μL)
0
20
40
60
80
100
120
60
73
101
145
185
249
300
Size (bp)
